# Four dimensions of naturalistic language production in aphasia after stroke

**DOI:** 10.1093/brain/awae195

**Published:** 2024-06-18

**Authors:** Marianne Casilio, Anna V Kasdan, Katherine Bryan, Kiiya Shibata, Sarah M Schneck, Deborah F Levy, Jillian L Entrup, Caitlin Onuscheck, Michael de Riesthal, Stephen M Wilson

**Affiliations:** Department of Hearing and Speech Sciences, Vanderbilt University Medical Center, Nashville, TN 37232, USA; Vanderbilt University Medical Center, Brain Institute, Nashville, TN 37232, USA; Department of Hearing and Speech Sciences, Vanderbilt University Medical Center, Nashville, TN 37232, USA; Department of Hearing and Speech Sciences, Vanderbilt University Medical Center, Nashville, TN 37232, USA; Department of Hearing and Speech Sciences, Vanderbilt University Medical Center, Nashville, TN 37232, USA; Department of Hearing and Speech Sciences, Vanderbilt University Medical Center, Nashville, TN 37232, USA; Department of Hearing and Speech Sciences, Vanderbilt University Medical Center, Nashville, TN 37232, USA; Department of Hearing and Speech Sciences, Vanderbilt University Medical Center, Nashville, TN 37232, USA; Department of Hearing and Speech Sciences, Vanderbilt University Medical Center, Nashville, TN 37232, USA; Department of Hearing and Speech Sciences, Vanderbilt University Medical Center, Nashville, TN 37232, USA; School of Health and Rehabilitation Sciences, University of Queensland, Brisbane QLD 4072, Australia

**Keywords:** connected speech, discourse, spoken language production, neuroimaging, neuroanatomy

## Abstract

There is a rich tradition of research on the neuroanatomical correlates of spoken language production in aphasia using constrained tasks (e.g. picture naming), which offer controlled insights into the distinct processes that govern speech and language (i.e. lexical-semantic access, morphosyntactic construction, phonological encoding, speech motor programming/execution). Yet these tasks do not necessarily reflect everyday language use. In contrast, naturalistic language production (also referred to as ‘connected speech’ or ‘discourse’) more closely approximates typical processing demands, requiring the dynamic integration of all aspects of speech and language. The brain bases of naturalistic language production remain relatively unknown, however, in part because of the difficulty in deriving features that are salient, quantifiable and interpretable relative to both speech-language processes and the extant literature. The present cross-sectional observational study seeks to address these challenges by leveraging a validated and comprehensive auditory-perceptual measurement system that yields four explanatory dimensions of performance—Paraphasia (misselection of words and sounds), Logopenia (paucity of words), Agrammatism (grammatical omissions) and Motor speech (impaired speech motor programming/execution).

We used this system to characterize naturalistic language production in a large and representative sample of individuals with acute post-stroke aphasia (*n* = 118). Scores on each of the four dimensions were correlated with lesion metrics, and multivariate associations among the dimensions and brain regions were then explored.

Our findings revealed distinct yet overlapping neuroanatomical correlates throughout the left-hemisphere language network. Paraphasia and logopenia were associated primarily with posterior regions, spanning both dorsal and ventral streams, which are critical for lexical-semantic access and phonological encoding. In contrast, agrammatism and motor speech were associated primarily with anterior regions of the dorsal stream that are involved in morphosyntactic construction and speech motor planning/execution, respectively.

Collectively, we view these results as constituting a brain–behaviour model of naturalistic language production in aphasia, aligning with both historical and contemporary accounts of the neurobiology of spoken language production.

## Teaser

Casilio *et al.* explore how everyday conversation skills are disrupted in aphasia during the first week after stroke. They find that distinct regions in the left hemisphere language network are specialized for different aspects of speaking, such as finding the correct word and producing speech sounds clearly.

## Introduction

There is a rich body of research on the neuroanatomical correlates of spoken language production in aphasia using constrained tasks such as picture naming and repetition.^1-15^ Although such tasks offer controlled insights into the processes underlying speech and language (i.e. lexical-semantic access, morphosyntactic construction, phonological encoding, speech motor planning/execution), performance does not necessarily reflect the dynamism of everyday language use.^16-19^ In contrast, naturalistic language production (also referred to as ‘discourse’ or ‘connected speech’)—the sequencing of words and utterances to convey meaningful messages^20^—requires the rapid integration of all aspects of speech and language.^21-25^ Yet despite being a closer approximation of typical speech-language processing demands, naturalistic language production is rarely used in studies of brain-behaviour relations in aphasia.

**Figure 1:**
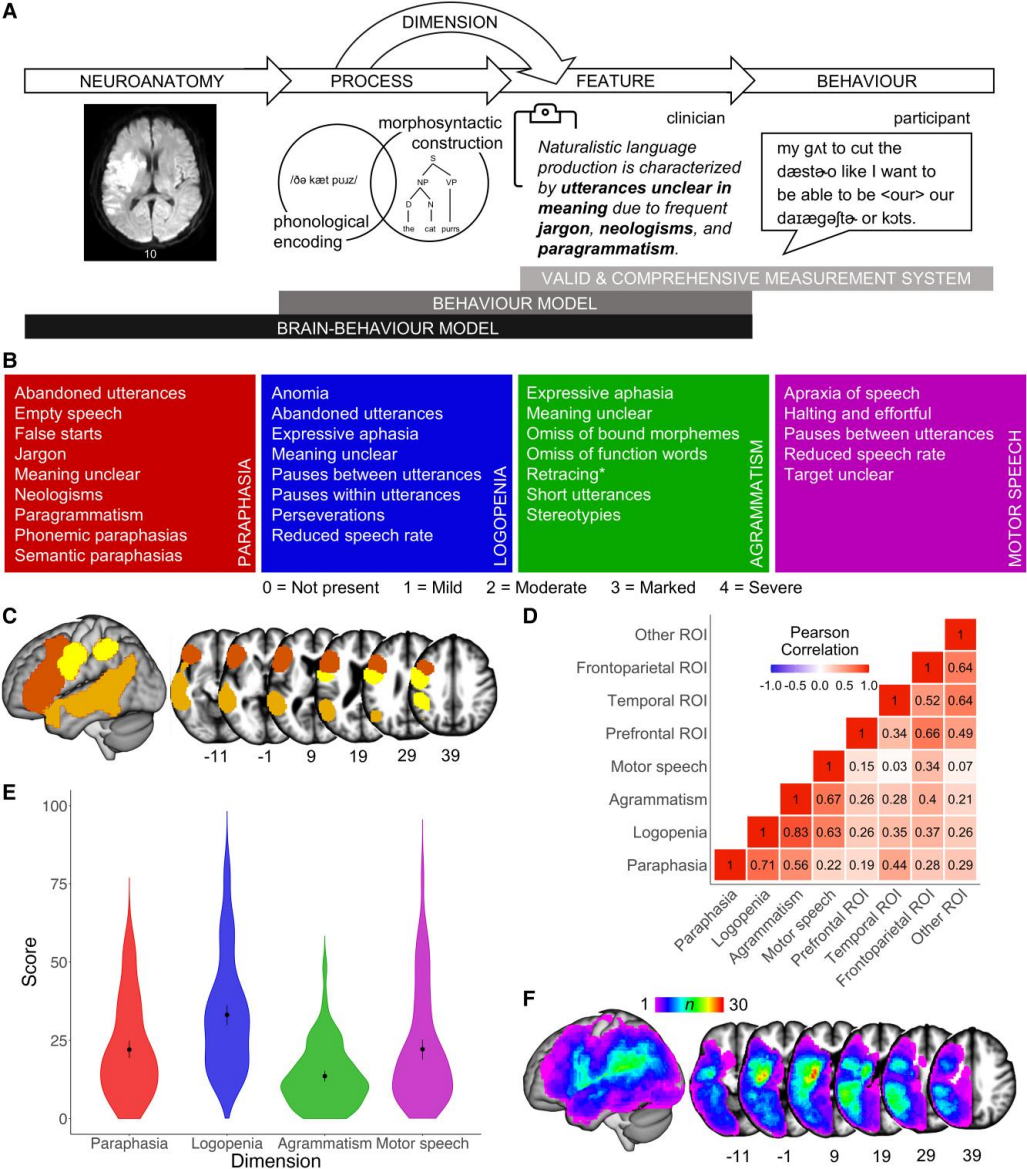
**Methodological details.** (**A**) Motivation and conceptual framework of the present study, showing the necessary stages in evaluating associations for understanding the neural correlates of naturalistic language production in aphasia. Behaviour–feature–process associations shown were addressed in our prior work.^25^ The following transcription conventions were used for displaying the language sample excerpt: < > indicate retracing; words transcribed using the international phonetic alphabet indicate phoneme-based errors (e.g. phonemic paraphasias, neologisms). Lesion and behavioural data are from Participant 1552. (**B**) Features that comprise the four dimensions of auditory-perceptual rating of connected speech in aphasia (APROCSA), each of which is shown in its own colour-coded box. Each feature is initially rated on the five-point scale shown directly below the boxes and then rescaled and averaged to create the dimension score; see main text for full scoring details. (**C**) Regions of interest (ROIs) displayed on a 3D surface rendering and representative axial slices of a template brain. The ROIs are defined as Prefrontal (dark orange); Frontoparietal (yellow); Temporal (light orange); and Other (not pictured), which encompasses all other areas within the left hemisphere. (**D**) Heat map depicting uncorrected Pearson correlations among the four ROIs and four APROCSA dimension scores. (**E**) Distribution of the four APROCSA dimension scores across the participants in the present study. The black dot indicates the mean while the black line shows the standard deviation. (**F**) Lesion distribution across study participants displayed on a three-dimensional surface rendering and representative axial slices of a template brain. As defined in the legend, the colour gradation corresponds to the number of participants with lesion overlap. *Score is expressed as a negative value per the factor analysis results (see Supplementary Table 1 for further details); bold = features identified from observed behaviours during a naturalistic language sample; omiss = omission.

Why is naturalistic language production an understudied area in aphasia? The answer is partially attributable to two methodological challenges. First, the range of observable behaviours during naturalistic language production (e.g. grammatical omissions, phonemic paraphasias) is highly diverse,^26-30^ yet there are few validated measurement systems (see Casilio *et al*.,^25^ Rochon *et al*.^31^ and Huber^32^) for operationalizing behaviours into a comprehensive and salient set of microstructural^21,22^ features. Second, even when features are quantified within a given system, there remains minimal understanding of how features relate to one another or underlying speech-language processes.^23-25^ Some features (e.g. phonemic paraphasia) have an apparent association with similar features (e.g. neologisms) and an underlying process (i.e. phonological encoding), while others (e.g. empty speech) lack such clear relations. In other words, to establish a brain–behaviour model of naturalistic language production in aphasia, one first needs a behaviour model of naturalistic language production in aphasia, and the development of that behaviour model depends on a comprehensive and validated measurement system (Fig. 1A).

Although the current literature has yielded preliminary insights into the neuroanatomical basis of naturalistic language production in aphasia,^4,9,33-57^ no study has addressed both of the challenges outlined here. One primary limitation has been the focus on individual features^9,33-44^ often presumed collectively to represent just one^9,34,35,43,44^ or two^36,38,40-42^ speech-language processes. Consequently, it is difficult to understand from this work how features and their underlying processes interact with one another, limiting the interpretation of any feature–brain associations. Others have pooled larger sets of features together, either *a priori* to obtain a unitary measure of impairment^45-48,56^ or *a posteriori* via data reduction techniques to identify across-feature commonalities that more clearly reflect an underlying process or anatomical region.^4,49-51,53-55,57^ However, the feature sets used were designed for subgroup classification,^4,45,48,53,54^ not comprehensive characterization; captured severity predominantly^54^; or again centred on one^49,57^ or two processes^50,55^; and many studies had relatively small sample sizes.^4,49-55,57^

In this cross-sectional observational study, we present a comprehensive account of brain-behaviour relations of naturalistic language production in aphasia after stroke. We address the aforementioned methodological challenges by leveraging our novel auditory-perceptual measurement system^25,58^—where a large set of impairment features is distilled into four explanatory dimensions of performance—and applying it to a large and representative group of individuals with aphasia^59^ who were tested acutely post-stroke. Continuous scores on each dimension are correlated with lesion metrics, and multivariate associations are explored at both behavioural and neural levels. Our resultant brain–behaviour model is then discussed relative to the extant literature on spoken language production and in the context of its practical utility for individuals with aphasia, their loved ones and their care teams.

## Materials and methods

### Participants

The present study included 118 participants with aphasia who were evaluated in the acute post-stroke period (1–5 days post-onset) and deemed to have a scorable naturalistic language sample, as defined later. Their demographic and medical history is presented in Table 1.

**Table 1:** Demographic and clinical characteristics of the participant sample

Age	64.1 ± 13.4 (21–87) years
Sex	72 male; 46 female
Handedness	105 right; 11 left; 2 ambidextrous
Race	97 White; 20 Black; 1 Native Hawaiian
Education	12.9 ± 3 (3–20) years
TOAST stroke type	
Large-artery atherosclerosis	21 (18%)
Cardioembolism	35 (30%)
Small-vessel occlusion	4 (3%)
Undetermined aetiology	36 (30%)
Haemorrhage	22 (19%)
Haemorrhagic transformation	17 (14%)
Lesion extent	34.3 ± 38.2 (0.6–261) cm^3^
Atrial fibrillation	26 (22%)
Coronary artery disease	25 (21%)
Cardiac arrythmia	15 (13%)
Diabetes mellitus	34 (29%)
Hyperlipidaemia	75 (64%)
Hypertension	98 (83%)
Myocardial infarction	11 (9%)
National Institutes of Health Stroke Scale on admission (0–42)^a^	7.5 ± 6.2 (0–25)
Days post-stroke on evaluation	2.9 ± 1.3 (1–6)
Quick Aphasia Battery^62^ (0–10)^a^	6.72 ± 1.68 (2.42–8.87)
Apraxia of speech^b^	12 (10%)
Dysarthria^b^	30 (25%)

^a^Higher scores denote greater impairment.

^b^Diagnoses were obtained by consensus agreement among a group of certified speech-language pathologists (authors M.C., S.M.S., J.L.E., C.O.) and experienced researchers (authors D.F.L., S.M.W.) using the Quick Aphasia Battery.^62^

Participant data was extracted from a larger longitudinal research study,^59^ where all individuals presenting to the Vanderbilt Stroke and Cerebrovascular Center at Vanderbilt University Medical Center between late 2016 and early 2020 were considered for inclusion. For this study, we recruited adults aged 18–90 years old who were premorbidly fluent in English with (i) acute ischaemic or haemorrhagic stroke to the left hemisphere or a clear presentation of aphasia following stroke to the right hemisphere; and (ii) infarct of at least 1 cm^3^, except thalamic infarcts of any volume and basal ganglia and/or white matter infarcts of at least ∼6 cm^3^ starting in late 2018. Individuals were not recruited if they had a (i) clinically unstable status or grave prognosis; (ii) previously symptomatic stroke; (iii) comorbid major psychiatric or neurological condition; or (iv) substance use disorder that interfered with study participation. Written informed consent was obtained from either the participant or their legally authorized representative, in accordance with the principles of the 1964 Declaration of Helsinki and with the approval of the Institutional Review Board at Vanderbilt University Medical Center.

As detailed in our prior work,^59^ 354 of a total 1055 individuals met inclusion criteria and consented to participate, 218 of whom received a clinical diagnosis of aphasia acutely. Given the present study’s focus, participants were only included if they additionally: (i) were testable acutely; (ii) had aphasia as assessed by both clinical impression and a score below the aphasia cut-off on the Quick Aphasia Battery (QAB; overall score of <8.9 out of 10)^60^; (iii) had a scorable naturalistic language sample; and (iv) had evidence that the stroke was to the non-dominant hemisphere. Whether a sample was scorable was determined based on prior criteria^24,25,60^ and operationalized as at least 3 min of participant speech or overt attempts at speech that were a minimum of 10 words per minute (per perceptual judgment of trained raters) and not exclusively characterized by phonological jargon, stereotypies or unintelligible productions. Of the 218 possible participants with aphasia, 118 were included in the present study. The other 100 participants were excluded for the following reasons: 18 were untestable acutely due to their medical status; 15 scored above the aphasia cut-off on the QAB; 1 showed clear clinical evidence of right-hemisphere language dominance (QAB overall score of 8.0 despite a total lesion volume of 248.4 cm^3^); and 66 did not have a scorable sample. The criteria for excluding the 66 participants without scorable samples were as follows: 22 had samples shorter than 3 min, 18 produced less than 10 words per minute; four produced only phonological jargon; three produced only stereotypies; seven produced only unintelligible speech; and 12 made no attempt at spoken language.

### Evaluation of naturalistic language production

#### Auditory-perceptual rating of connected speech in aphasia

Naturalistic language production was quantified using auditory-perceptual rating of connected speech in aphasia (APROCSA),^25,58^ a psychometrically robust and comprehensive measurement system validated on a diverse sample of individuals with chronic aphasia.^25^ Based on the gold-standard auditory-perceptual approach for evaluating neuromotor speech disorders,^61-63^ APROCSA involves rating more than twenty salient features on a five-point scale, where higher scores denote greater impairment. It is both efficient and accessible, requiring no transcription or specialized software/equipment. Beyond possessing strong interrater agreement, APROCSA’s features correlated with several constrained language tasks and transcription-based naturalistic language measures, and per a factor analysis yielded four explanatory dimensions^25^: Paraphasia, Logopenia, Agrammatism and Motor speech. We collectively view these findings as constituting a behaviour model that links observed features of naturalistic language production with speech-language processes via four dimensions, as described below.

### Paraphasia

Defined as the misselection of words and sounds, this dimension is characterized by phonemic, morphosyntactic and semantic errors that are often self-corrected. Features salient to the Paraphasia dimension correlated strongly with analogous measures derived from transcription.^25^ Within the classical taxonomy, Paraphasia reflects fluent output^47,64,65^; however, when contextualized within psychological models of spoken language production,^66-69^ the Paraphasia dimension reflects one way that lexical-semantic access and phonological encoding may be impaired, whereby competing representations are erroneously retrieved and encoded, leading to selection errors. Paraphasia may potentially also reflect a selection-specific disruption in morphosyntactic construction (i.e. paragrammatism) distinct from the Agrammatism dimension described below, although the processes that drive both paragrammatic and agrammatic output are subject to ongoing debate.^70-72^

### Logopenia

Drawing on its historical definition, paucity of words, this dimension is marked by prominent anomia that leads to effortful, slow and abandoned utterances. Features salient to the Logopenia dimension, which reflect one facet of non-fluency as per the classical taxonomy,^47,64,73^ correlated strongly with not only similar transcription-based measures but also overall severity and naming performance on constrained tasks.^25^ When considered relative to psychological models of spoken language production,^66-69^ the Logopenia dimension reflects another way that the same processes of the Paraphasia dimension (lexical-semantic access, phonological encoding) are affected. In contrast to Paraphasia, the Logopenia dimension reflects degraded access to word and sound representations^74,75^ and/or strategic suppression of would-be selection errors,^76,77^ which leads to impoverished output or so-called errors of omission (e.g. ‘I can’t think of the word’).^74^

### Agrammatism

A well-established construct in the aphasia literature,^78,79^ this dimension’s definition mirrors its classical interpretation: grammatical omissions. Here, utterances are short and simplified, often stereotyped, and lacking in morphosyntactic structures (e.g. bound morphemes). Features salient to the Agrammatism dimension, which reflect another facet of non-fluency, correlated strongly with analogous transcription measures.^25^ Within the same modelling framework described for the previous two dimensions, the Agrammatism dimension likely reflects impaired morphosyntactic processing, although speculation persists regarding the underlying nature of agrammatic output.^71,72^

### Motor speech

The Motor speech dimension incorporates impairment in speech motor programming (apraxia of speech) and/or execution (dysarthria). It is characterized by distinctive features, such as distorted articulation and the presence of a concomitant apraxia of speech, but also features that co-occur with aphasia, such as effortful and slow speech production. Features salient to the Motor speech dimension, which reflect yet another facet of non-fluency, correlated strongly with not only analogous transcription measures but also measures of phonemic errors (e.g. rate of neologisms), the latter of which are rarely distinguished from motoric errors in other naturalistic language measurement systems.^80,81^

### Dimension scoring

To obtain participant scores on each of the four dimensions, we created a novel scoring system (Supplementary Fig. 1), which was based on a modification of the factor analysis from our prior work^25^ conducted on 24 participants chronically post-stroke (Supplementary Table 1). Specifically, we used the factor loading matrix from this modified analysis to select sets of features to be included in the four dimensions scores for the present study. A loading cut-off of >|0.4| was chosen based on a careful review of all available data, where the aim was to include the maximal number of features for reliability purposes while also only retain features viewed as critical to the underlying factors per our prior work^25^ and that of others.^23,37^ This yielded nine features for the Paraphasia dimension, eight for the Logopenia dimension, seven for the Agrammatism dimension and five for the Motor speech dimension (Fig. 1B). We then used an established rating scale scoring system^82^ to derive four dimension scores, where feature ratings for a given dimension are transformed to a 0–100 scale (0 = 0, 1 = 25, 2 = 50, 3 = 75, 4 = 100) and then averaged. Unlike factor or component scores commonly used in studies on the neuroanatomical correlates of speech-language processing in aphasia,^1-4,52,53^ this type of scoring system can be readily reproduced by other researchers and implemented in clinical settings.

### Procedures

#### Sample elicitation

All participants completed an audiovisual-recorded speech-language evaluation with a certified speech-language pathologist (authors S.M.S., J.L.E., C.O.) using the QAB,^60^ an efficient yet comprehensive aphasia battery. The QAB includes a naturalistic language sample elicitation protocol, which was used for the present study. In this protocol, participants engage in conversational speech with the clinician for at least 3 min through answering a series of semi-structured interview questions (e.g. ‘describe what you like about where you live’ or ‘tell me what brought you to the hospital’). Unlike other commonly used elicitation proctols,^80,83^ active interaction between the participant and the clinician is encouraged. The protocol mimics a typical clinical encounter, where a provider often aims to establish rapport and obtain pertinent background information.

#### Sample preparation

To control for potential length effects, audiovisual recordings were clipped to contain the first 3 min of participant speech or overt attempts at speech. This included times when participants may have used multiple modalities for communication, although periods where a person exclusively used a modality other than speaking (e.g. pointing to images) were not counted towards the 3-min sample length. Although a minimum sample length of 5 min was used in our previous APROCSA work,^25,58^ this was adjusted given the truncated length of the sample elicitation protocol in the QAB.

#### Scorers

Three certified speech-language pathologists (authors M.C., K.B., K.S.) independently reviewed the samples. All had extensive expertise in aphasia and language analysis and were blinded as far as possible to other available data (e.g. behavioural testing results, neuroimaging data). Author M.C. was the primary scorer, reviewing all 118 samples. Authors K.B. and K.S. served as secondary scorers, each reviewing a pseudo-randomly selected subset of 20% of the participants (*n* = 23). All scorers reviewed the same participant subset but each in a pseudo-randomized order. Both secondary scorers underwent a standardized training program before scoring.^84^ Aside from evaluating interrater agreement, as described below, scores from the primary scorer were used for all analyses.

#### Scoring procedure

Following our published guidelines,^25^ samples were watched twice and scored in real time without pausing the video. Notetaking and pausing between the two viewings to review scores was encouraged, although scorers were instructed to spend no more than 15 min per sample and to limit scoring to 1 h sessions to reduce listener fatigue.^85^ As with the elicitation protocol, the scoring procedure was designed to simulate time and technological constraints commonplace in clinical settings and that are known barriers to implementing naturalistic language production assessment in aphasia.^86^

### Neuroimaging

#### Image acquisition

As described previously,^59^ we obtained MRI or CT studies that were acquired as part of routine clinical care, either at Vanderbilt or an outside hospital. After reviewing all available imaging within the first 30 days after stroke, we selected the first study where the lesion was visible and stable in size; preference was given to MRI over CT images when available. Of the 118 participants, MRI was selected for 99 participants and CT was selected for 19 participants.

#### Lesion delineation and image processing

Lesions were manually delineated in native space on diffusion weighted imaging (DWI) for ischaemic infarcts, fluid attenuated inverse recovery (FLAIR) for haemorrhagic infarcts or CT by trained research staff using ITK-SNAP^87^ following previously defined procedures.^59^ Other available image sequences within a selected study, co-registered to the native image sequence, were referenced as needed. After lesion delineation, the image sequence used for subsequent processing (FLAIR for MRI, CT for CT) was co-registered to the lesion mask, if needed, and warped to Montreal Neurological Institute (MNI) space using Unified Segmentation^88^ and enantiomorphic normalization,^89^ whereby the right hemisphere was used as a mirror image to ‘fill in’ lesioned tissue of the left hemisphere. Once processed, the normalized lesion masks were smoothed using an 8 mm full-width at half-maximum Gaussian filter. All image processing was carried out using MATLAB 2019a and SPM12.^90^

#### Regions of interest

Regions of interest (ROIs) were constructed (Fig. 1C) to support a subset of the analyses of the present study. Our aim in creating these ROIs was to identify key neuroanatomical regions that are functionally specific to the left-hemisphere language network.^91-95^ To do this, we used activation patterns from healthy speakers performing two adaptive functional MRI paradigms: a semantic judgment paradigm^91^ and a pseudoword rhyme judgment paradigm.^92^ This combination of paradigms was selected for their excellent clinical sensitivity to critical regions supporting all speech-language processes.^93^ Thus, the activation patterns from these paradigms, although not highly localized to a specific speech-language process, provide a useful starting point for defining broad brain regions likely important to naturalistic language production in aphasia.

Four ROIs spanning the entire left hemisphere were specified using group-level maps from the two paradigms (*n* = 16; voxel-wise threshold: *P* < 0.005; corrected at *P* < 0.05 based on cluster extent using permutation testing).^91,92^ The first, Prefrontal, included regions involved in morphosyntactic processing^96-101^ and linguistic control.^102^ The Prefrontal ROI was defined as activations greater than zero on the semantic judgment paradigm in voxels in the following regions of the Automated Anatomical Labelling (AAL) atlas^103^: 1 (left precentral gyrus), 7 (left middle frontal gyrus), 11 (left pars opercularis), 13 (left pars triangularis) and 15 (left part orbitalis). The second ROI, Frontoparietal, included regions typically implicated in phonological encoding^104-106^ and speech motor planning/execution,^107,108^ and was defined as activations greater than zero on the rhyme judgment paradigm but not semantic judgment paradigm in AAL regions 1 (left precentral gyrus), 57 (left postcentral gyrus), 61 (left inferior parietal lobe) and 63 (left supramarginal gyrus). The third ROI, Temporal, included regions important to all language processes^109,110^ and was defined as activations greater than zero on the semantic judgement paradigm in AAL regions 55 (left fusiform gyrus), 65 (left angular gyrus), 81 (left superior temporal gyrus), 83 (left superior temporal pole), 85 (left middle temporal gyrus), 87 (left middle temporal pole) and 89 (left inferior temporal gyrus). The fourth ROI, Other, included all regions within the left hemisphere that fell outside the three aforementioned ROIs. The Other ROI was included to not only account for any potential effects of overall lesion extent but also to capture potential associations in regions beyond the left-hemisphere language network, as naturalistic language production may involve other aspects of cognitive processing.^111^

Given that functional activation is restricted to grey matter yet stroke lesions commonly also involve underlying white matter, we dilated the first three ROIs using a full-width half-maximum Gaussian filter (4 mm for the Prefrontal and Temporal; 6 mm for Frontoparietal, as the rhyme judgment paradigm was relatively less sensitive^92^), followed by a cut-off that prevented spatial overlap among the ROIs. We then calculated lesion volume (cm^3^) within the four ROIs for each participant.

### Statistical analysis

Prior to our main analyses, we quantified interrater agreement between the primary scorer and the secondary scorers for ratings on the 23 APROCSA features that comprise the dimensions^25^ for the 23 participant subset. Here, we used intraclass correlation coefficients of absolute agreement with random effects for both participants and scorers^112^ with bootstrapped 95% confidence intervals (CIs) calculated with a bias-corrected accelerated method^113,114^ from 10 000 iterations of resampled data (of note, all bootstrapped 95% CIs of the present study were calculated using this method). Analyses were completed using R 4.3.0. Results were interpreted qualitatively as poor (*r* < 0.40), fair (0.40 ≤ *r* < 0.60), good (0.60 ≤ *r* < 0.75) or excellent (*r* ≥ 0.75) following established guidelines.^115^ Across all features and participants, agreement between the primary scorer and each secondary scorer was good [M.C.–K.B.: *r* = 0.61, 95% CI (0.55, 0.66)] or nearly good [M.C.–K.S.: *r* = 0.59, 95% CI (0.51, 0.65)]. Agreement overall was similar to our prior work^25^ yet modestly reduced likely due to inevitable additional confounds (e.g. lethargy) present in individuals with aphasia in the acute post-stroke period.

#### Characterizing the APROCSA dimensions

To validate our novel scoring system, we evaluated each dimension’s internal consistency, or the degree to which a measure captures the construct(s) of interest. Specifically, feature**–**dimension associations were evaluated qualitatively via bivariate scatterplots and further quantified via (i) polyserial correlations with bootstrapped 95% CIs^113,114^; and (ii) McDonald’s ω_t_,^116^ a reliability coefficient appropriate for multivariate measures, for the dimension scores. Analyses were completed using R 4.3.0.

#### Mapping the APROCSA dimensions to lesions

To establish associations between structural brain damage and each dimension individually, complementary lesion-symptom mapping analyses were completed at two levels of neural specificity—ROI and voxel-wise. In the case of the ROI analyses, this allowed us to investigate brain-behaviour relations in broad regions functionally specific to the left-hemisphere language network, and to statistically account for the known interdependency among brain regions^117^ (Fig. 1D). For the voxel-wise analyses, this permitted us to obtain a fine-grained and anatomically agnostic understanding of brain-behaviour associations.

To explore associations between structural brain damage and all four dimensions simultaneously, we conducted a series of multivariate ROI and voxel-wise analyses. This was to account for the known interdependency among the APROCSA dimensions (Fig. 1D) and to quantify the unique association between a particular brain region and a given APROCSA dimension when controlling for the influence of the other three dimensions. We specifically were interested in understanding which regions were unique to a given dimension and which ones were shared across dimensions.

### Dimension–ROI associations

We specified four multiple linear regression models to quantify the effect of lesion volume in all four ROIs (Prefrontal, Frontoparietal, Temporal, Other) on each of the dimension scores. Statistical significance of an ROI’s effect on a dimension score was determined using a two-tailed *t*-test at *P* < 0.05. Cohen’s *f*^2^ effect sizes, which reflect the variability explained by an ROI that otherwise is not accounted for in the remaining ROIs, were additionally reported and interpreted qualitatively as small (0.02 ≤ *f*^2^ < 0.15), medium (0.15 ≤ *f*^2^ < 0.35) and large (*f*^2^ ≥ 0.35) following established guidelines.^118^ Analyses were completed using R 4.3.0.

### Dimension–voxel-wise associations

Using a mass univariate approach spanning the whole brain,^37,119^ we specified four sets of multiple linear regression models to quantify the effect of lesion status in individual voxels on each of the dimension scores. Only voxels lesioned in at least ∼5% of participants (*n* = 6) were considered to avoid spurious associations.^120^ Total lesion extent was included as a controlling covariate. Statistical significance of a voxel’s effect on a dimension score was determined using a one-tailed (alternative hypothesis: *β* > 0) *t*-test of *P* < 0.01. Voxels with a significant effect were retained, and the size of contiguous clusters of significant voxels was recorded. Statistical significance for clusters was then determined using permutation testing with 10 000 iterations, where the behavioural data was randomly shuffled and associated with the lesion masks; only clusters with sizes falling within the extreme 5% of the permuted null distribution’s right tail were considered significant (i.e. corrected *P* < 0.05). The *t*-statistics for the permutation-corrected clusters were then plotted. Analyses were carried out using MATLAB 2019a.

### Multivariate associations

For the ROI analyses, we evaluated the effect of an individual ROI on the multivariate distribution of all four dimension scores simultaneously, allowing us to confirm that any significant associations identified were above and beyond any behavioural interdependencies. This was done using a two-tailed *F*-test at *P* < 0.05 from Pillai’s trace,^121^ a statistic estimated within a multivariate analysis of variance framework and one that reflects a predictor’s effect on all outcome variables simultaneously.

For the voxel-wise analyses, we first evaluated the degree of spatial overlap among the permutation-corrected clusters to determine whether there were significant associations in the same brain region for two or more APROCSA dimensions. This was done by calculating Dice–Sorensen coefficients (DSC),^122,123^ a generalized agreement metric^124,125^ commonly used in neuroimaging research,^91,92^ with bootstrapped 95% CIs^113,114^ for each pair of dimensions (e.g. Paraphasia–Logopenia overlap). The DSC was also averaged across all pairwise maps to evaluate the multivariate spatial overlap in all four dimensions. As with the intraclass correlation coefficients, results were interpreted qualitatively.^115^ Spatial overlap and non-overlap between pairs of maps and among all four maps were also plotted.

For pairwise maps with non-negligible spatial overlap (i.e. DSC or bootstrapped 95% CI ≥ 0.4, or in the fair range or better), we then explored the relative magnitude of brain-behaviour associations within the area of overlap. Our aim here was to evaluate the degree to which a significant association between an APROCSA dimension score and a given voxel was above and beyond that of an association between another dimension score in the same voxel. We conducted this analysis using a voxel-wise bias metric. Specifically, *t-*statistics from the two permutation-corrected maps were standardized to a 0–100 scale, constrained to include only voxels overlapping in both maps, and the difference between the standardized *t-*statistics of the two maps in each voxel was then calculated and plotted. These plotted bias values, which can range from 0–100, reflect the magnitude of rank differences in statistical significance within overlapping voxels for two permutation-corrected clusters in either direction. The more extreme (i.e. farther from zero) a bias value in a given voxel, the greater the difference in statistical significance between the two maps despite their spatial overlap. The less extreme (i.e. closer to zero) a bias value in a given voxel, the smaller the difference in statistical significance between the two maps. Analyses were completed using MATLAB 2019a and R 4.3.0.

## Results

### Characterizing the APROCSA dimensions

**Figure 2:**
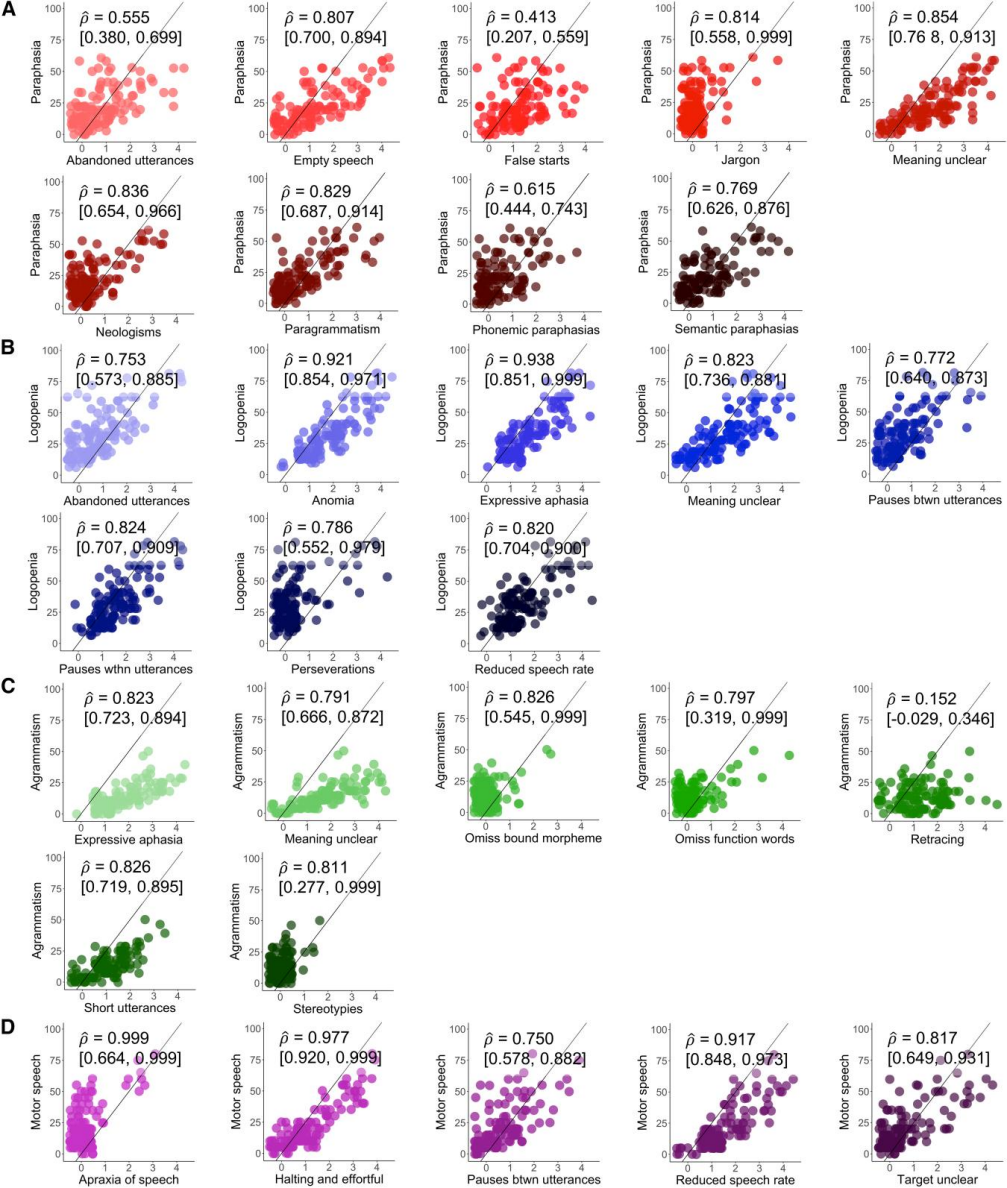
**Feature–dimension associations.** Bivariate scatterplots showing the association among dimension scores (*y*-axis) for (**A**) Paraphasia, (**B**) Logopenia, (**C**) Agrammatism and (**D**) Motor speech and their individual feature ratings (*x*-axis). The black 45-degree line serves as a reference point for interpreting the relative proximity of feature ratings to the dimension scores. Plotted points were randomly jittered to increase visibility of individual feature ratings. Polyserial correlation coefficients ($\hat{\rho }$) and bootstrapped 95% confidence intervals (CIs) of the feature–dimension associations are reported at the *top left*. btw = between; omiss = omission; wthn = within.

A wide spread of individual differences was appreciable for both the dimension scores (Fig. 1E) and the feature–dimension associations (Fig. 2), again demonstrating that APROCSA comprehensively captures salient features of naturalistic language production in aphasia.^25^ There also was converging evidence of high internal consistency (i.e. alignment between observed scores and their intended constructs) among the features that comprised each of the dimension scores. The polyserial correlations were strong; 26 of 29 possible bivariate associations were $\hat{\rho }$ > 0.7 with generally tight bootstrapped 95% CIs (Fig. 2). McDonald’s *ω_t_* coefficients were similarly high (Paraphasia: *ω_t_* = 0.87; Logopenia: *ω_t_* = 0.92; Agrammatism: *ω_t_* = 0.89; Motor speech: *ω_t_* = 0.87).

### Mapping the APROCSA dimensions to lesions

The lesion distribution of the participants spanned the left middle cerebral artery territory (Fig. 1F), as to be expected in a post-stroke aphasia sample. The maximum overlap was in the left posterior insula (MNI coordinates: *x* = −36, *y* = −19, *z* = 3). Both the ROI and voxel-wise analyses yielded highly comparable results despite statistical differences in their neural granularity and consideration of interdependency among brain regions, thus providing converging evidence on the neuroanatomical correlates of the four APROCSA dimensions. As such, findings from both analyses are reported according to APROCSA dimension, followed by a description of the multivariate associations among the four APROCSA dimensions.

#### Paraphasia

**Figure 3:**
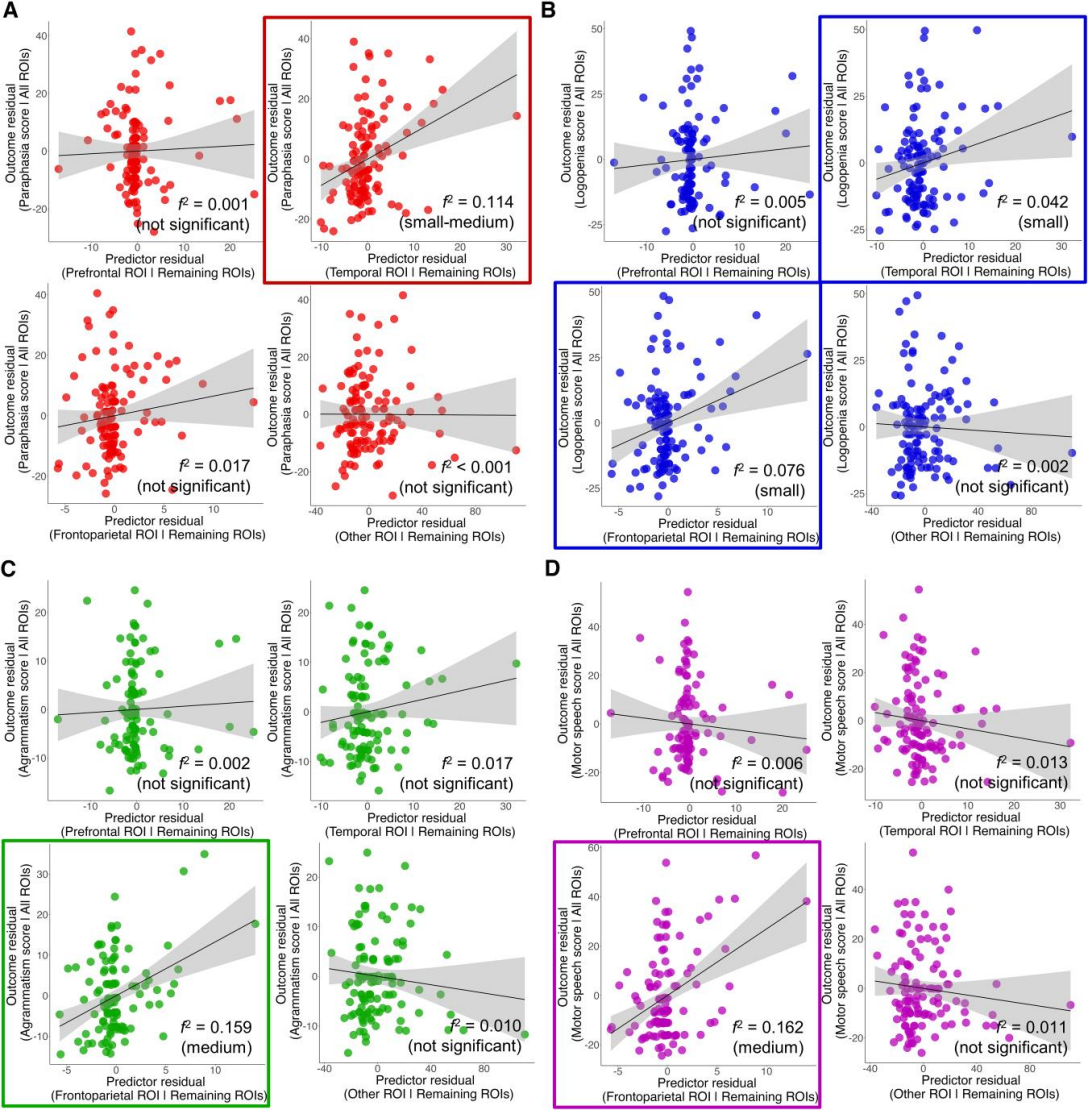
**Dimension–ROI associations.** Partial regression plots depicting the magnitude and directionality of the effect of each region of interest (ROI) on the (**A**) Paraphasia, (**B**) Logopenia, (**C**) Agrammatism and (**D**) Motor speech dimension scores of APROCSA. The *y*-axes reflect the residuals of a model where the dimension score is regressed onto all the ROIs (i.e. ‘All ROIs’ in the axis title) except the ROI of interest. The *x*-axis shows the residuals of a model where the ROI of interest is regressed onto the remaining ROIs (i.e. ‘Remaining ROIs’ in the axis title). The solid black trend line reflects the slope estimate of an ROI for the model, and the grey band displays the slope estimate’s 95% confidence interval (CI). The association between the *x*- and *y*-axes reflects the partial correlation between an APROCSA dimension and a given ROI. When squared, this is equivalent to a Cohen’s *f*^2^ effect size, which is reported along with its qualitative interpretation in the *bottom-right* corner of the plots, and reflects the variability in an APROCSA dimension explained by an ROI that is not accounted for in the remaining ROIs. Plots outlined in a colour-coded box indicate ROIs that were significantly associated with a dimension. The full results are listed in Table 2.

**Figure 4:**
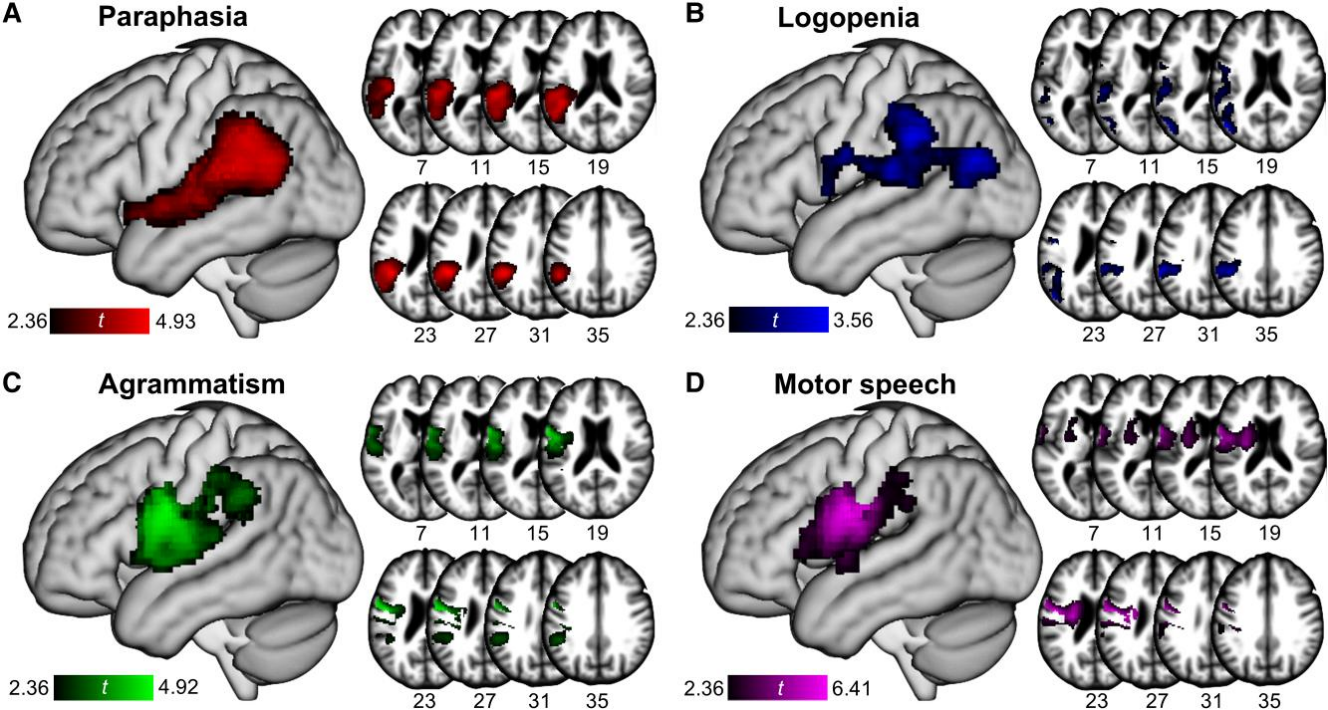
**Dimension–voxel-wise associations.** Permutation-corrected significant clusters on a three-dimensional surface rendering and representative axial slices of a template brain for the (**A**) Paraphasia, (**B**) Logopenia, (**C**) Agrammatism and (**D**) Motor speech dimension scores. As defined in the legend, the colour gradation of the cluster corresponds to the range of significant *t-*statistics in the analysis. The full methodological details and results of the voxel-wise analyses can be found in the main text.

Scores on the Paraphasia dimension were significantly associated with predominantly left temporal and, to a lesser extent, left parietal regions (Figs 3A and 4A and Table 2). For the ROI analysis, there was a significant effect of the Temporal ROI (*P* < 0.001; Table 2), which explained ∼11% of variability in Paraphasia scores not accounted for by the remaining ROIs (Fig. 3A). Individuals with more damage in the Temporal ROI had greater impairment on the Paraphasia dimension on average when holding damage in the remaining ROIs constant. The Prefrontal, Frontoparietal and Other ROIs were not significant predictors.

**Table 2:** Effect of the four ROIs on the univariate distribution of a given APROCSA dimension score

ROI	*β*	SE	*t*	*P*
**Paraphasia^a^**				
(Intercept)	17.573	1.683	10.441	<0.001
Prefrontal	0.090	0.239	0.378	0.706
Frontoparietal	0.652	0.469	1.389	0.167
Temporal	0.869	0.228	3.817	<0.001
Other	−0.003	0.060	−0.048	0.961
**Logopenia^a^**				
(Intercept)	28.273	2.014	14.041	<0.001
Prefrontal	0.206	0.286	0.720	0.473
Frontoparietal	1.713	0.561	3.053	0.003
Temporal	0.606	0.272	2.225	0.028
Other	−0.954	2.025	−0.471	0.639
**Agrammatism^a^**				
(Intercept)	11.389	1.096	10.391	<0.001
Prefrontal	0.065	0.155	0.420	0.675
Frontoparietal	1.330	0.305	4.353	<0.001
Temporal	0.210	0.148	1.419	0.159
Other	−0.042	0.039	−1.082	0.281
**Motor speech^a^**				
(Intercept)	22.345	2.068	10.321	<0.001
Prefrontal	−0.244	0.293	−0.833	0.406
Frontoparietal	2.702	0.576	4.687	<0.001
Temporal	−0.335	0.280	−1.198	0.233
Other	−0.082	0.074	−1.114	0.268

APROCSA = Auditory-Perceptual Rating Of Connected Speech In Aphasia; ROI = region of interest; SE = standard error.

^a^Paraphasia model fit: *F*(4,113) = 9.524, *P* < 0.001, *R*^2^ = 0.252; Logopenia model fit: *F*(4,113) = 9.508, *P* < 0.001, *R*^2^ = 0.252; Agrammatism model fit: *F*(4,113) = 10.562; *P* < 0.001, *R*^2^ = 0.272; Motor speech model fit: *F*(4,113) = 6.237, *P* < 0.001, *R*^2^ = 0.181.

For the voxel-wise analysis, one large and significant cluster emerged (volume = 51.30 cm^3^; maximum *t* = 4.93; corrected *P* < 0.001), spanning almost the entirety of posterior temporoparietal cortex and extending into underlying white matter (Fig. 4A). With a peak in left supramarginal gyrus (MNI coordinates: *x* = −44, *y* = −42, *z* = 28), the cluster encompassed nearly all the left middle and posterior superior temporal gyrus and sulcus and the posterior middle temporal gyrus, traversing dorsally into angular and supramarginal gyri and anteroinferiorly into the mid-portion of the superior temporal gyrus with complete involvement of Heschl’s gyri and adjacent white matter. The cluster also extended inferomedially into the external capsule; internal capsule; superior longitudinal fasciculus, including the arcuate; and edges of the thalamic radiation and corona radiata.

#### Logopenia

Scores on the Logopenia dimension were significantly associated with predominantly left parietal and, to a lesser extent, posterior temporal and frontal regions (Figs 3B and 4B and Table 2). For the ROI analysis, there were significant effects of both the Frontoparietal ROI (*P* = 0.003; Table 2) and the Temporal ROI (*P* = 0.03). The effect of the Frontoparietal ROI was greater, explaining ∼8% of variability in Logopenia scores not accounted for by the remaining ROIs; the Temporal ROI explained ∼4% of variability (Fig. 3B). Individuals with more damage in the Frontoparietal ROI and, to a lesser extent, the Temporal ROI had greater impairment on the Logopenia dimension when holding damage in the remaining ROIs constant. The Prefrontal and Other ROIs were not significant predictors.

For the voxel-wise analysis, one significant cluster emerged (volume = 22.05 cm^3^; maximum *t* = 3.56; corrected *P* = 0.014), encompassing most of the left inferior parietal lobe, including supramarginal and angular gyri (Fig. 4B). With a peak in left middle temporal gyrus (MNI coordinates: *x* = −52, *y* = −72, *z* = 20), the cluster also extended anteriorly into the left ventral precentral gyrus, Rolandic operculum and pars opercularis; posteroinferiorly into the middle occipital lobe; and medially into the superior longitudinal fasciculus, including the arcuate.

#### Agrammatism

Scores on the Agrammatism dimension were significantly associated with predominantly left frontal regions (Figs 3C and 4C and Table 2). For the ROI analysis, there was a significant effect of the Frontoparietal ROI (*P* < 0.001; Table 2), which explained ∼16% of variability in Agrammatism scores not accounted for by the remaining ROIs (Fig. 3C). Individuals with more damage to the Frontoparietal ROI had greater impairment on the Agrammatism dimension when holding damage in the remaining ROIs constant. The Prefrontal, Temporal and Other ROIs were not significant predictors.

For the voxel-wise analysis, one significant cluster emerged (volume = 30.15 cm^3^; maximum *t* = 4.92; corrected *P* = 0.005, involving the majority of the left precentral gyrus (Fig. 4C). The peak of the cluster was also in this area (MNI coordinates: *x* = −60, *y* = 4, *z* = 22), although the cluster extended anteroinferiorly into the frontal operculum, dorsally into the Rolandic operculum and medially into the superior longitudinal fasciculus and superior corona radiata. Notably, the ventral edge of the cluster reached the superior temporal lobe, ranging from pole to midpoint and into left Heschl’s gyri.

#### Motor speech

Scores on the Motor speech dimension were significantly associated with predominantly left frontal and subcortical regions (Figs 3D and 4D and Table 2). For the ROI analysis, there was a significant effect of the Frontoparietal ROI (*P* < 0.001; Table 2), which explained ∼16% of variability in Motor speech scores not accounted for by the remaining ROIs (Fig. 3D). Individuals with more damage in the Frontoparietal ROI had greater impairment on the Motor speech dimension when holding damage in the remaining ROIs constant. The Prefrontal, Temporal and Other ROIs were not significant predictors.

For the voxel-wise analysis, one significant cluster emerged (volume = 28.29 cm^3^; maximum *t* = 6.41; corrected *P* = 0.006, spanning much of the left ventral precentral gyrus (Fig. 4D). The peak of the cluster was in this area (MNI coordinates: *x* = −48, *y* = −8, *z* = 26) but also descended inferomedially into the insula, caudate, putamen and underlying white matter, including the internal capsule, external capsule and corona radiata. There additionally was a small extension of the cluster dorsally into the left inferior parietal lobe.

#### Multivariate associations

For the ROI analyses, Pillai’s trace was significant for the Temporal (*P* < 0.001), Frontoparietal (*P* < 0.001) and Prefrontal (*P* = 0.002) ROIs; the Other ROI was not significant (Supplementary Table 2). In other words, the Temporal, Frontoparietal and Prefrontal ROIs were significantly associated with all four dimension scores simultaneously. Thus, the significant associations for both the Temporal and Frontoparietal ROIs, as detailed above, are robust to the inter-correlation among the four APROCSA dimension scores. Importantly, Pillai’s trace only provides information about the inclusion of a predictor; additional information about those predictors (e.g. magnitude of association) comes from the four previously described univariate models.

**Figure 5:**
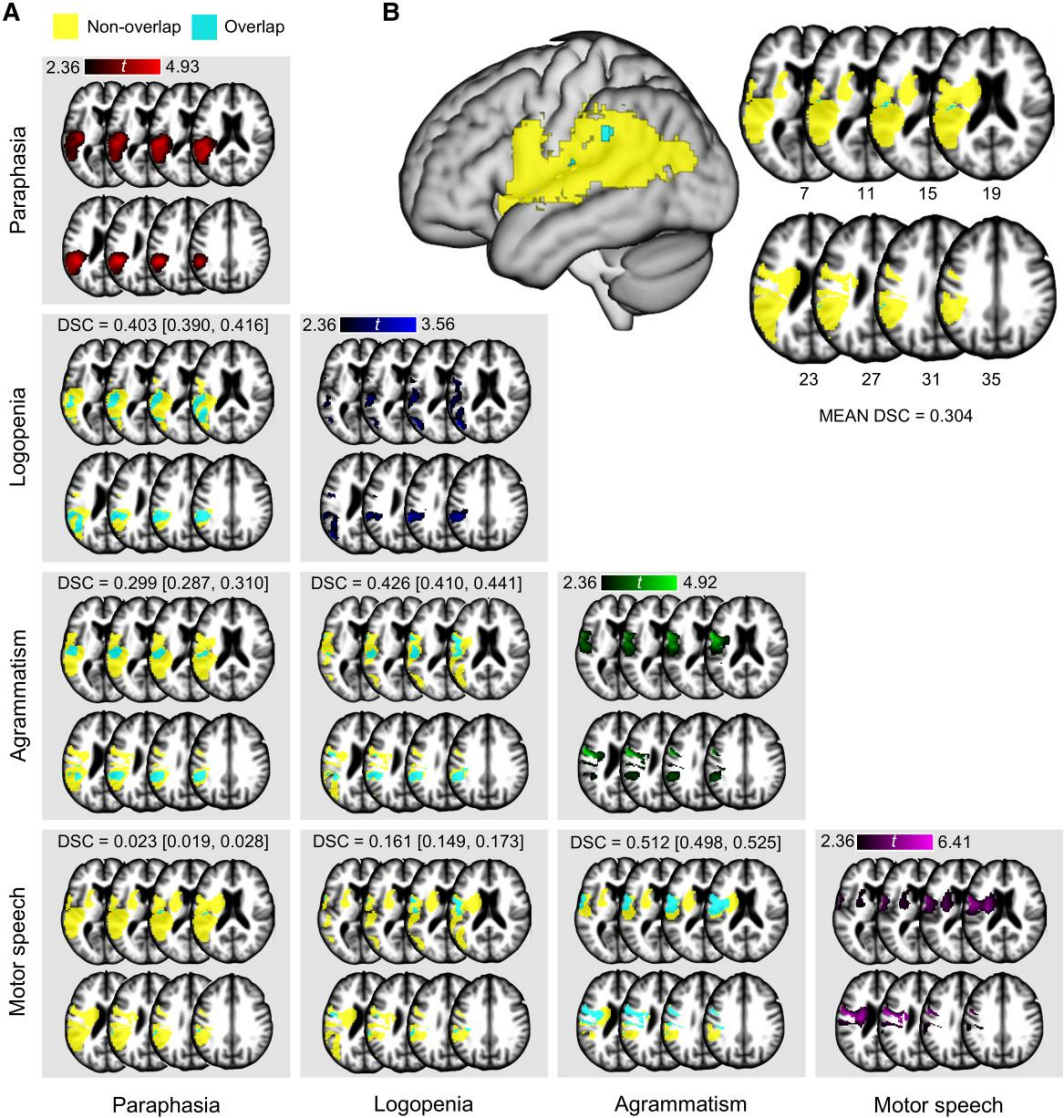
**Multivariate voxel-wise spatial overlap.** (**A**) Matrix of representative axial slices (from *left* to *right*, *top* to *bottom*: *z* = 7, 11, 15, 19, 23, 27, 31, 35; note these are the same slices shown in Fig. 4) that depict the spatial overlap (cyan) and non-overlap (yellow) for voxel-wise lesion correlates between pairwise maps of the four APROCSA dimensions. Dice–Sorenson Coefficients (DSC) and their bootstrapped 95% confidence intervals are also shown. (**B**) Representative axial slices and a three-dimensional surface rendering that show the spatial overlap (cyan) and non-overlap (yellow) among all maps of the four APROCSA dimension scores, as well as the mean DSC across all possible pairwise combinations.

For the voxel-wise analyses, several unique regions were identified for each of the four dimensions (Fig. 5A). This included the majority of the left posterior superior temporal gyrus and sulcus for the Paraphasia dimension. For the Logopenia dimension, unique regions were portions of left supramarginal gyrus, posterior middle temporal gyrus and temporal-occipital junction. The Agrammatism dimension was uniquely associated with portions of left Rolandic operculum while the Motor speech dimension was uniquely associated with the left basal ganglia.

In line with the above findings, all but three APROCSA dimension pairs displayed *poor* spatial overlap (Fig. 5A). There were small amounts of overlap in the left Rolandic operculum (Paraphasia–Agrammatism, Paraphasia–Motor speech, Logopenia–Agrammatism, Logopenia–Motor speech), left superior temporal cortex (Paraphasia–Agrammatism, Logopenia–Agrammatism) and left inferior parietal lobe (Paraphasia–Motor speech, Logopenia–Motor speech). The multivariate spatial distribution, or the spatial overlap among all pairs of maps simultaneously, was also poor, with only a marginal amount of overlap in the left Rolandic operculum and supramarginal gyrus (Fig. 5B).

There was fair spatial pairwise overlap for the Paraphasia–Logopenia [DSC = 0.403, 95% CI (0.390, 0.416)], Logopenia–Agrammatism [DSC = 0.426, 95% CI (0.410, 0.441)] and Agrammatism–Motor speech [DSC = 0.512, 95% CI (0.498, 0.525)] maps (Fig. 5A). Overlap for Paraphasia–Logopenia primarily encompassed portions of left posterior temporal and inferior parietal cortex. For Logopenia–Agrammatism, overlap was also in left inferior parietal cortex, as well as ventral precentral gyrus, Rolandic operculum and pars opercularis. For Agrammatism–Motor speech, overlap was concentrated in left ventral precentral gyrus and underlying white matter.

**Figure 6:**
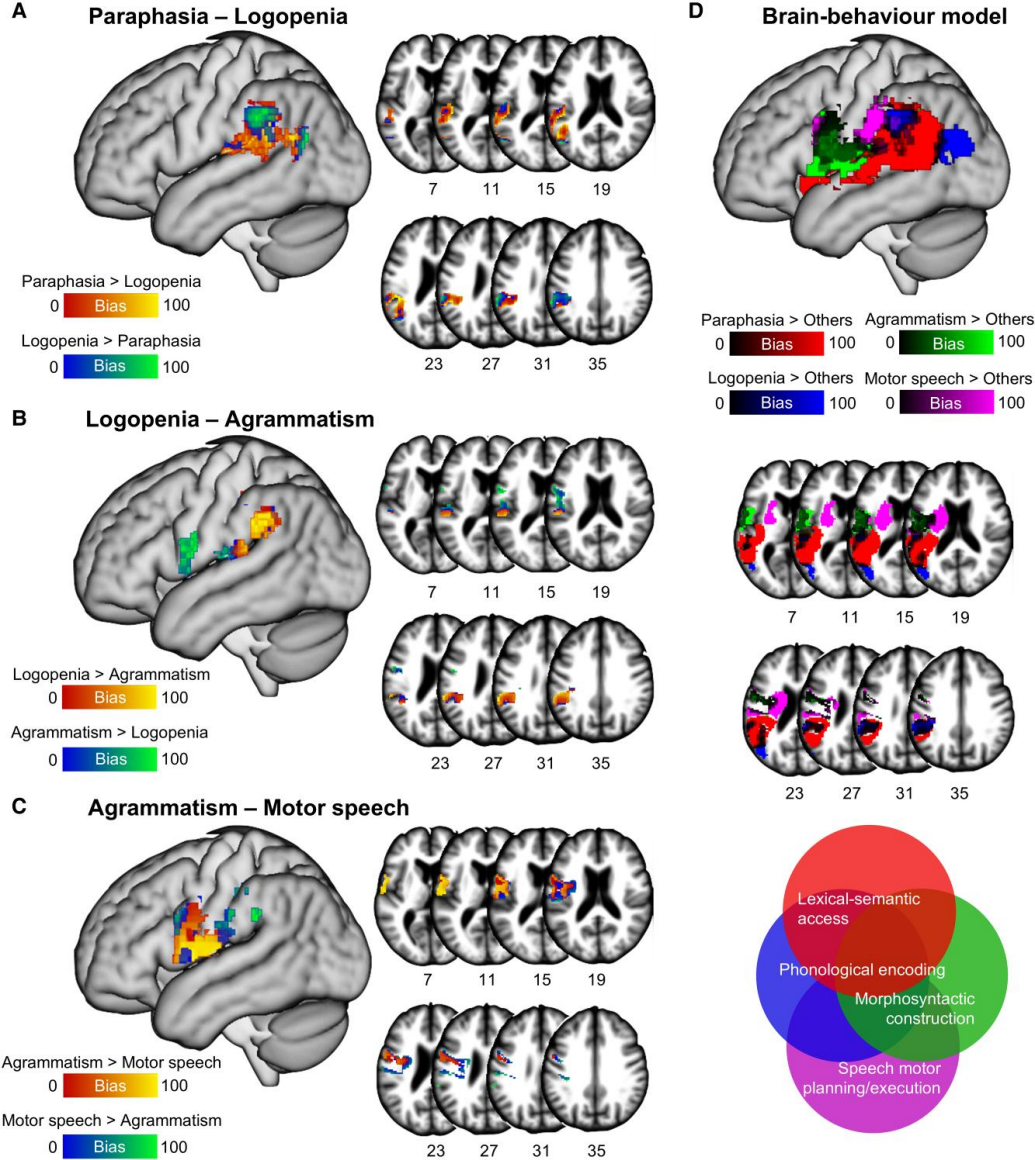
**Multivariate associations among the four APROCSA dimensions.** Bias maps, displayed on three-dimensional surface renderings and representative axial slices of a template brain, between the (**A**) Paraphasia–Logopenia, (**B**) Logopenia–Agrammatism and (**C**) Agrammatism–Motor speech dimensions. As defined in the legend, the colour gradation corresponds to the magnitude of bias in either direction, as described in the main text. Methodological details and additional findings are described in the main text and Fig. 5. Panel **D** shows our proposed brain–behaviour model of naturalistic language production in aphasia. The *top* portion shows findings displayed on a three-dimensional surface rendering and representative axial slices of a template brain show the bias maps with the unique permutation-corrected cluster correlates for each dimension (i.e. associated only with that dimension and not the other three) reincorporated and scaled to have a maximal bias value (i.e. 100). For dimensions with two bias values (Logopenia, Agrammatism), values were averaged. The bottom portion depicts a construct map, where each semi-opaque circle represents a dimension and within each dimension are the speech-language processes implicated, positioned with respect to their relative influence on one or more dimensions. Notably, consistent with the model, all dimensions are overlapping and all processes are positioned within more than one dimension, reflecting the inter-correlations at both levels.

When the three pairs of maps with non-negligible overlap (Paraphasia–Logopenia, Agrammatism–Motor speech) were explored in greater detail, a bias gradient emerged both within and across left posterior temporoparietal and left frontal regions. For Paraphasia–Logopenia (Fig. 6A), *t-*statistics were greater for the Paraphasia dimension for the majority of the overlap region, peaking in left posterior superior temporal gyrus (MNI coordinates: *x* = −51, *y* = −36, *z* = 16) and adjacent left superior temporal sulcus. In contrast, *t-*statistics were greater for the Logopenia dimension in the left supramarginal gyrus (MNI coordinates: *x* = −61, *y* = −37, *z* = 32) and in the left middle temporal gyrus, peaking in a location near-identical to the univariate map of the latter region (MNI coordinates: *x* = −57, *y* = −67, *z* = 18). For Logopenia–Agrammatism (Fig. 6B), *t*-statistics were greater for the Logopenia dimension in most of the left inferior parietal lobe, peaking in the left supramarginal gyrus (MNI coordinates: *x* = −61, *y* = 38, *z* = 30). Greater *t*-statistics for the Agrammatism dimension were instead in the left ventral precentral gyrus and Rolandic operculum, peaking in the former region (MNI coordinates: *x* = −52, *y* = −3, *z* = 27). For Agrammatism–Motor speech (Fig. 6C), *t-*statistics that were greater for the Agrammatism dimension were in near-identical areas to those of Logopenia–Agrammatism, encompassing most of the left ventral precentral gyrus and left Rolandic operculum and peaking in the latter region (MNI coordinates: *x* = −60, *y* = 1, *z* = 5). Greater *t-*statistics for the Motor speech dimension were in left ventral precentral gyrus and underlying white matter, as well as inferior parietal lobe. Peaks were in both left ventral precentral gyrus (MNI coordinates: *x* = −41, *y* = −6, *z* = 36) and supramarginal gyrus (MNI coordinates: *x* = −60, *y* = −36, *z* = 32).

## Discussion

The purpose of the present study was to characterize the neuroanatomical basis of naturalistic language production in aphasia using a robust measurement system and in the context of established underlying speech-language processes. Our results, which were derived from a large and representative sample of individuals tested in the acute post-stroke period, revealed that four dimensions of naturalistic language production were differentially and systematically associated with distinct brain regions across the left-hemisphere language network. These dimensions not only possessed high internal consistency but also showed sensitivity to a range of individual differences (Figs 1E and 2), a finding not previously demonstrated among naturalistic language measures used in prior studies^9,33-56^ and one that bolsters the interpretability and generalizability of the main neuroanatomical findings.

In the discussion that follows, the neuroanatomical correlates of the four dimensions are each interpreted in the context of the behaviour model outlined above and relative to the extant literature. Of particular interest was the degree to which our findings were congruent with the historical connectionist model,^73,126-128^ where a fundamental anterior–posterior distinction is aligned with a fluency dichotomy,^47^ and its contemporary derivation, the dual stream model,^3,129-131^ in which speech-language processing more generally is subdivided into dorsal and ventral streams.

### Dimensions with posterior neuroanatomical correlates involving both dorsal and ventral streams

#### Paraphasia

Defined by a variety of selection errors (Fig. 2A), the unique neuroanatomical correlate of the Paraphasia dimension was left superior temporal gyrus and sulcus, as per the multivariate voxel-wise analyses (Fig. 6A). The voxel-wise analyses revealed involvement of other regions, most notably left supramarginal gyrus, left angular gyrus and left posterior middle temporal gyrus (Fig. 4A). The ROI analysis implicated the same superior temporal regions; the Temporal ROI was the only significant predictor (Fig. 3A and Table 2).

Nearly all connectionist accounts converge on left superior temporal cortex, a posterior region, as critical for spoken language production. First described as the seat of ‘acoustic imagery’^65^ by Karl Wernicke, associations between this region and the selection of phonological, lexical and even morphosyntactic representations span the history of the field. Multiple early autopsy case series identified left superior temporal cortex as important not only in the production of neologistic jargon aphasia,^132^ but also, depending on the subregion, in phonemic, semantic and paragrammatic misselection.^133^ Both historical and contemporary *in vivo* imaging studies showed similar results, with post-Rolandic lesions most commonly resulting in fluent aphasia syndromes^132-136^ that were characterized primarily by frequent selection errors.^64,137,138^

In the dual stream model,^3,129,130,139^ which draws heavily on psychological frameworks of speech-language processing,^68,69,140^ left superior temporal cortex is a critical region situated at the origin of both the dorsal and ventral processing streams (see Hickok and Poeppel^130^) and thus is responsible for the integration of acoustic, phonological and semantic representations. Recent studies have linked left superior temporal cortex and underlying white matter damage to impaired performance on metrics derived from constrained tasks, including composite scores indexing both phonological encoding^1,4,52,57,141^ and lexical-semantic access,^8^ as well as a range of paraphasia types.^8,38,142^ For naturalistic language production specifically, left superior temporal cortex damage has been associated with features salient to the Paraphasia dimension—paragrammatism^34^ or reduced syntactic structure,^33^ phonological paraphasias^37^ or reduced phonematic structure,^33^ increased false starts and repaired sequences^37^ and reduced type-token ratio^41,52^ (akin to APROCSA’s ‘Empty speech’ feature)—and composite metrics of overall complexity.^53,54^

#### Logopenia

Defined by a variety of omissive errors (Fig. 2B), the unique neuroanatomical correlates of the Logopenia dimension were left supramarginal gyrus, posterior middle temporal gyrus and temporal-occipital junction, as per the multivariate voxel-wise analyses (Figs 6A and B). The voxel-wise analysis revealed the involvement of other regions, predominantly left angular gyrus, posterior superior temporal gyrus, ventral precentral gyrus, Rolandic operculum and pars opercularis (Fig. 4B). The ROI analysis similarly implicated multiple regions; both the Frontoparietal and Temporal ROIs were significant predictors (Fig. 3B and Table 2).

Left supramarginal gyrus and left inferior parietal lobe are critical to neo-connectionism, where these posterior regions are responsible for cross-modal associations for a variety of cognitive functions relevant to spoken language production.^127,128,143,144^ Ascribing to the connectionist account of Norman Geschwind,^127^ contemporary Frank Benson was equivocal on whether lesions to this posterior region resulted in fluent versus non-fluent aphasia syndromes.^47,134^ Although initial anatomical evidence found this region to be most associated with fluent aphasia,^47^ subsequent autopsy and *in vivo* imaging studies have revealed that persistent Broca’s aphasia, a non-fluent syndrome, is necessitated not on left frontal damage but the additional involvement of left inferior parietal lobe and its underlying white matter.^46,48,59,145,146^ Benson and others additionally showed that two anomia syndromes (word production, semantic)^134,147^ were localized to left inferior parietal lobe, inferring that damage to this association relay station had the capacity to disrupt access to semantic, lexical and phonological representations. Indeed, left inferior parietal lobe has been strongly implicated in repetition tasks,^5,9,33,148-151^ which likely involve all aspects of speech-language processing to varying degrees. Moreover, many of these studies have implicated adjacent regions also important to the Logopenia dimension, specifically left posterior middle temporal gyrus, left temporal-occipital junction and underlying white matter.^45,134,152,153^

In the context of the dual stream model, left supramarginal gyrus, a dorsal stream region involved in sound-to-articulation mappings,^3,129,130,139^ has been associated with neologistic^7^ and thematic semantic errors^6^ in picture naming, as well as composite metrics of phonological encoding derived from similar constrained tasks.^2,4,51,52,141,154,155^ In many of these studies, left frontal regions of the dorsal stream are also implicated in composite metrics of not only phonological encoding^4,141,154,155^ but also lexical-semantic access.^2,141^ Left posterior middle temporal gyrus, a ventral stream region involved in sound-to-meaning mappings^129,130,139^ and temporal-occipital cortex have instead been associated with omissive errors in picture naming^156^ and composite metrics of lexical-semantic access obtained from constrained tasks.^51,52^ For naturalistic language production specifically, supramarginal and middle temporal gyri and underlying white matter have been associated with features that define the sparse output of the Logopenia dimension, including decreased maximal speech rate,^37^ increased pauses within utterances^44^ and decreased nouns produced per minute.^49^ Multiple composite metrics of naturalistic language production have also been associated with these regions, all of which broadly index the quantity^49,52,53,57^ and complexity^49,50,53,54^ of words and utterances produced. Importantly, left frontal regions have similarly been implicated in indices of both quantity^51,52^ and quality.^51^

### Dimensions with anterior neuroanatomical correlates involving the dorsal stream

#### Agrammatism

Defined by short and simplified utterances containing grammatical omissions (Fig. 2C), the unique neuroanatomical correlates of the Agrammatism dimension were left ventral precentral gyrus and left Rolandic operculum, as per the multivariate voxel-wise analyses (Figs 6B and C). The voxel-wise analysis revealed the involvement of other regions, including underlying white matter and subcortical structures (Fig. 4C). The ROI analysis yielded congruent results; the Frontoparietal ROI was the only significant predictor (Fig. 3C). The Prefrontal ROI, which contains left inferior frontal gyrus, was not significant in the ROI analysis, nor was there a significant association with this region in the voxel-wise analysis.

There is a long yet contentious history on the nature of agrammatic output within the connectionist framework, although the vast majority of evidence localizes its features to the left frontal lobe, an anterior region. Defined by Adolf Kussmaul^157^ but perhaps more canonically associated with Paul Broca,^158,159^ the motor aphasia syndrome now known as Broca’s aphasia and putatively associated with agrammatism was described as a destruction to ‘motor imagery’^65^ by early connectionists. In the accounts that followed, it was found that lesions to left ventral precentral gyrus, an anterior region, were the most common locus of the non-fluent aphasia syndromes,^47^ of which Broca’s aphasia was prevalent.^138^ This accords with historic and recent observations that morphosyntactic deficits resolve rapidly following circumscribed damage to left inferior frontal gyrus (Broca’s area),^46,56,59,145,146,160,161^ the classically held locus of agrammatism, and suggests the importance of left parietal regions (see Logopenia discussion above) and other frontal regions, especially left ventral precentral gyrus and the Rolandic operculum.^56^

Understanding agrammatic output in the context of the dual stream model is not straightforward. There remains considerable ongoing debate regarding the underlying nature of agrammatic output and whether it is distinct from paragrammatism,^72,162^ restricted to a single modality,^163-166^ or instead reflective of strategic suppression, whereby redundant information is omitted to boost communicative salience and efficiency.^71,72,167,168^ The dual stream model appears to support the view of strategic suppression, as ventral precentral gyrus is a dorsal stream region important to articulatory, not morphosyntactic, processing.^130^ However, other empirical evidence does suggest the important role of morphosyntactic construction in agrammatic output; in studies of naturalistic language production, several features comparable to those in the Agrammatism dimension have been associated with left ventral precentral gyrus and left Rolandic operculum, including decreased speech rate,^37,41^ overall tokens,^41,52^ number of embeddings,^37^ and verbs per minute^40^; and perceptual classification of agrammatic output.^9,34,35,163^ Composite metrics of naturalistic language production that index sentence accuracy,^53^ complexity,^49^ and severity^37,49,57^ have also been associated with these regions.

#### Motor speech

Defined by effortful and slow productions with articulatory errors (Fig. 2D), the unique neuroanatomical correlates of the Motor speech dimension were left basal ganglia and adjacent white matter (Fig. 6C). The voxel-wise analysis revealed the involvement of other regions, most prominently left ventral precentral gyrus (Fig. 4D). The ROI analysis identified only the Frontoparietal ROI as a significant predictor (Fig. 3D), which suggests the importance of left ventral precentral gyrus; however, this finding may be secondary to the absence of any subcortical ROIs.

The importance of left basal ganglia and adjacent white matter, both anterior regions, in speech motor execution is virtually unchallenged in the literature and in a tradition largely distinct from connectionism and others. Although there existed early conceptualizations of neuromotor speech disorders,^169,170^ systematic characterization did not begin until the mid-20th century.^61,62,171^ Here, bilateral basal ganglia damage was first identified as critical to dysarthrias characterized by hypo- or hyperkinetic movement, as identified on a motor speech examination.^62^ Subsequent research found unilateral damage to motor regions, including basal ganglia, was sufficient to result in dysarthria,^63,172,173^ although such disruptions were found to be limited to the acute post-stroke period (i.e. the same timeframe of the present study).^59^ For speech motor planning, the association between left ventral precentral gyrus and apraxia of speech has been well-established after stroke^12,63,174-176^ and in neurodegeneration.^10,177^ In line with this prior work, left ventral precentral gyrus would likely be the primary correlate of the Motor speech dimension in a sample of individuals chronically post-stroke, at which point apraxia of speech is typically more common than dysarthria.^59^ In contrast, dysarthria was more common in the present study (Table 1), a finding also observed in other studies on individuals in the acute post-stroke period,^178,179^ and its relative prevalence is likely driving the current findings.

### Towards a brain–behaviour model of naturalistic language production in aphasia

We argue that neuroanatomical findings from our four APROCSA dimensions constitute an initial yet comprehensive brain–behaviour model of naturalistic language production in aphasia, with clear links to psychological models and neuroanatomical evidence on speech-language processing (Fig. 6D). Given the broad congruence of our findings with the literature on a range of aphasia etiologies, we anticipate this model is generalizable beyond stroke but with some important caveats (e.g. our model in its present form does not include the role of the left anterior temporal lobe in pre-lexical anomia evidenced in semantic variant primary progressive aphasia^180-182^).

In this model, disruption to lexical-semantic access and phonological encoding is primarily captured by the Paraphasia and Logopenia dimensions, albeit in distinct ways. As detailed above, the two dimensions converge on damage to predominantly posterior regions in the left temporal and parietal lobes known to be critical for both speech-language processes. The two dimensions also diverged in neuroanatomically meaningful ways, as revealed in detail by the multivariate voxel-wise analyses and bolstered by the broad findings from the ROI analyses. Specifically, Paraphasia most strongly localized to left superior temporal gyrus and sulcus—regions shown to be important to the selection of semantic, lexical and phonemic forms. In contrast, Logopenia most strongly localized to left supramarginal gyrus, left middle temporal gyrus and left temporal-occipital junctions—regions also associated with access to analogous forms. As detailed above in their respective sections of the Discussion, this distinction between access and selection in lexical-semantic access and phonological encoding can be understood within both a connectionist and dual-stream framework; however, other accounts are additionally congruent. Within the context of semantic cognition,^183^ both the Paraphasia and Logopenia dimensions reflect a problem with the control system, where linguistic forms are known but cannot be reliably selected in appropriate contexts.^184^ Disruption to this system, which relies on a wide network of left frontal, temporal and parietal regions,^185,186^ could result in the selection of erroneous competing representations, as is the case for the Paraphasia dimension, or in the inability to select any representation, competitor or not, as observed in the Logopenia dimension. Notably, the Logopenia dimension was associated with multiple regions in left frontal cortex (Fig. 4B), albeit not uniquely so when other dimensions were accounted for (Fig. 6B).

In contrast to other studies using constrained tasks,^1,2^ we did not identify any clear distinction, behaviourally or neurally, between lexical-semantic access and phonological encoding. Although ultimately multifactorial (e.g. typical lesion distribution in aphasia after stroke), one key difference between prior work and the present study is differences in how behaviours manifest during naturalistic language production versus constrained tasks; for example, paraphasias produced during a language sample are not clearly associated with performance on picture naming.^134,187^ This difference in performance may be related to strategic suppression during naturalistic language production, where errors can potentially be avoided when a specific target word or phrase is not required. Notably, strategic suppression of would-be selection errors is thought to potentially subserve omissive errors in constrained tasks^74,77^ and has been directly observed in a prior report of individuals with left parietal damage^134^; thus, it cannot be entirely ruled out as an alternative explanation for the access deficit that defines the Logopenia dimension.

Disruption to morphosyntactic construction is predominantly captured by the Agrammatism dimension, although the Paraphasia dimension also plays a non-trivial role with regard to the selection of morphosyntactic markers.^9,34,72^ As detailed above, the Agrammatism dimension is associated with left ventral precentral gyrus, an anterior region that is less commonly reported in relation to morphosyntactic construction. Notably, left ventral precentral gyrus, along with left Rolandic operculum and pars opercularis, were neuroanatomical correlates shared with the Logopenia dimension. Although a clear anterior–posterior gradient was present (Fig. 6B), both dimensions are characterized by omissive errors that are either lexical (Logopenia) or morphosyntactic (Agrammatism) in nature, and this commonality cannot be readily understood within either the connectionist or dual stream frameworks, where agrammatism is not consistently defined or explained. When viewed through the lens of semantic cognition, however, Logopenia and Agrammatism both reflect a problem with the control system, as defined above, and an inability to select lexical and morphosyntactic representations respectively due to damage to left frontal and parietal regions.

Disruption to speech motor programming/execution is captured by the Motor speech dimension. This dimension was most uniquely localized to left basal ganglia and white matter pathways, both of which are classical regions for multiple dysarthria subtypes. Motor speech also converged with agrammatism in its association with left ventral precentral gyrus, a region frequently associated with apraxia of speech.

A few important conclusions emerge from this model. First, core dimensions of naturalistic language production in aphasia are anatomically distinct, even when accounting for known inter-correlations. Our voxel-wise analyses identified non-negligible spatial overlap for only three pairs of maps (Fig. 5), which were shown to dissociate in magnitude when explored in greater depth (Fig. 6A–C). From a clinical standpoint, this speaks to the importance of deeply considering anatomical findings in conjunction with behavioural assessment, as associations among the APROCSA dimensions may be readily disentangled when considered relative to lesion information. From a neuroscientific standpoint, this supports the view of speech and language functions arising from the concerted interplay of distinct and specialized brain regions,^110,188^ even in naturalistic contexts and where inter-correlations are modelled instead of constrained.

Despite these distinctions, there are also regions of overlap. Specifically, left inferior frontal and inferior parietal regions are shared across multiple APROCSA dimensions, with Logopenia and Agrammatism being the most notable. This suggests an important role for the dorsal stream, irrespective of the connectionist anterior-posterior division, in executing a range of speech-language processes important to naturalistic language production. It also could suggest that dorsal stream regions within the ‘control’ system of semantic cognition play a critical role here. Both left prefrontal^189^ and left inferior parietal^190^ regions have been independently identified as critical to this system, and it may be that both, in addition to other left frontal regions (e.g. ventral precentral gyrus), must operate synergistically to meet the task demands of naturalistic language production, where representations are generally more complex and the need to integrate cross-modal information (e.g. gesture) is far greater.

Second, overall lesion extent—particularly in areas outside the left-hemisphere language network—plays an ancillary role in naturalistic language production in aphasia in the acute post-stroke period. The voxel-wise analyses only implicated regions within the language network, a finding that was somewhat expected given that overall lesion extent was included as a controlling covariate (Fig. 4). We also identified no role for the Other ROI, which indexed damage to all other non-language regions of the left hemisphere, in any of the univariate and multivariate ROI analyses (Fig. 3, Table 2 and Supplementary Table 2). This was despite the fact that ∼92% of participants had more damage to non-language regions than language regions. In other words, a relatively small amount of damage within the language network was sufficient to disrupt naturalistic language production in the acute post-stroke period, and the nature of the disruption was distinct to the specific region of damage. This finding speaks not only to the sensitivity of naturalistic language production^16,18,21,134^ but also to the value of investigating the brain basis of speech and language in the acute period of stroke recovery.^148,191^

Third, the brain basis of naturalistic language production—as measured with APROCSA—appears scalable from constrained speech-language tasks. We identified no clear role of brain regions beyond the language network, and those regions we identified within the language network were comparable to those reported in studies of picture naming, repetition and other tasks.^1-15^ Critically, our four dimension scores yielded meaningful neuroanatomical correlates across the left-hemisphere language network from a brief assessment of naturalistic language production, which not only mirrors everyday language use^16-19^ but also is highly aligned with the rehabilitation priorities^192,193^ and self-reported functional communication^194^ of individuals with aphasia. Thus, the findings of the present study raise a new and important question: Why not study naturalistic language production in aphasia?

### Clinical applications

It is our view that the four dimensions of APROCSA and their neuroanatomical correlates are a rich source of information relevant to individuals with aphasia, their loved ones and their care teams. The findings presented here could be used to provide education, monitor recovery trajectories and design behavioural interventions. To this end, we have created an accessible version of this article for individuals with aphasia and their loved ones (Supplementary Document 1).

Materials supporting the implementation of APROCSA are available online (see ‘Data availability’ statement for links), including a structured training program,^84^ freely sharable video examples^58^ and a score calculator. APROCSA is a reliable measurement system created for use within the typical constraints of clinical practice. Thus, scores obtained by a provider during an encounter can be interpreted in direct relation to this study and with reference to routine clinical neuroimaging, which we used for the brain-based analyses. In other words, all aspects of the present study were purposefully designed to be useful for those who provide care to individuals with aphasia.

### Limitations

The present study has several noteworthy limitations. First, individuals with unscorable naturalistic language samples were excluded. This was a necessary constraint; however, it consequently means that our sample was skewed towards participants with less severe speech-language impairments and with lesions in particular regions, thus likely influencing the brain associations we identified. In particular, we did not have extensive coverage in more anterior left frontal regions (Fig. 1F), as individuals with large lesions to these regions are often not testable acutely.^59^ In the future, we plan to test whether APROCSA dimension scores can be reasonably predicted based on other acute factors (e.g. constrained task scores, lesion information).

Second, APROCSA and its four dimensions focus exclusively on the microstructural features of naturalistic language production (i.e. operationalized behaviours that occur at the utterance level or below).^21,22^ Our focus on microstructural features is and has been a pragmatic one, as supra-utterance macrostructural features (e.g. coherence) tend to have relatively poorer psychometric properties^22^ and do not map neatly onto speech-language processes.^187^ Nonetheless, naturalistic language production requires the integration of microstructure and macrostructure,^22,111,195^ and both should be emphasized in future research.^196^ Importantly, the inclusion of macrostructural features in non-stroke populations with aphasia, where structural damage is not restricted by vascular territory, will likely be critical in revealing the role of regions beyond the left-hemisphere language network (e.g. hippocampus) in naturalistic language production.^197^

Third, although our dimension scoring system showed high internal consistency, a confirmatory factor analysis conducted in a new sample of participants in the chronic post-stroke period (analogous to our original factor analysis^25^) would provide additional evidence supporting the generalizability of the four dimensions comprising APROCSA.^117^

Fourth, we did not include any measures of cognition, which may be useful in disentangling the influence of other relevant processes (e.g. executive function^4,51,57^) and would be a valuable future direction.

Finally, our ROI and voxel-wise analyses concentrate primarily on cortical and, to a lesser extent, subcortical grey matter despite the potential important role of white matter.^141,151,198-201^ Our methods, which remain a predominant approach for evaluating brain–behaviour relations,^202^ yield some information about white matter; however, follow-up studies should include imaging modalities that assess white matter integrity (e.g. diffusion tensor imaging) to build on the model presented here.

## Conclusion

We have shown that four dimensions of naturalistic language production in aphasia are associated with distinct yet overlapping regions within the left-hemisphere language network in the acute post-stroke period. These findings are aligned with both historical and contemporary accounts of the neurobiology of spoken language production, refining and expanding upon our understanding of the mechanisms that drive the dynamic use of speech and language in naturalistic contexts.

## Supplementary Material

awae195_Supplementary_Data

## Data Availability

Data and code for the present study, as well as resources to support the implementation of APROCSA, are available at https://github.com/mcasilio/neuroaprocsa. An interactive website including the individual lesion masks, clinical neuroimaging, and audiovisual naturalistic language samples from the larger study dataset will be made available at a future date at: https://langneurosci.org/recovery.
