# Supplementary material for: Four dimensions of naturalistic language production in aphasia after stroke
Source: Brain. 2024 Jun 18;148(1):291–312. doi: 10.1093/brain/awae195 (PMC11706289; doi:10.1093/brain/awae195)
Supplement: awae195_Supplementary_Data [file awae195_supplementary_data.pdf]

Supplementary Materials for Casilio *et al.* 'Four dimensions of naturalistic language production in aphasia after stroke'

Supplementary Table 1 Factor loading matrix for APROCSA from a modified analysis conducted on data from Casilio *et al.*[1]

	Oblimin-rotated standardized factor loadings				
Feature	Paraphasia (22% variance)	Logopenia (21% variance)	Agrammatism (20% variance)	Motor speech (17% variance)	Communality
Anomia	0.28	0.79	0.23	0.03	0.94
Abandoned utterances	0.51	0.65	-0.20	0.12	0.88
Empty speech	0.73	0.31	-0.08	-0.38	0.78
Semantic paraphasias	0.57	0.15	-0.38	-0.10	0.56
Phonemic paraphasias	0.94	-0.09	0.08	-0.07	0.84
Neologisms	0.66	0.09	0.31	0.25	0.68
Jargon	0.81	0.01	0.04	0.25	0.74
Perseverations	-0.23	0.91	-0.10	-0.08	0.72
Stereotypies and automatisms	0.15	0.26	0.67	0.02	0.63
Short and simplified utterances	-0.12	0.30	0.72	0.26	0.95
Omission of bound morphemes	0.05	-0.11	0.98	-0.05	0.90
Omission of function words	-0.05	-0.06	0.98	0.03	0.97
Paragrammatism	0.75	-0.18	-0.11	-0.06	0.59
Pauses between utterances	-0.23	0.61	0.02	0.47	0.78
Pauses within utterances	-0.10	0.69	0.05	0.34	0.76
Halting and effortful	0.02	0.30	0.06	0.78	0.91
Reduced speech rate	-0.24	0.46	0.19	0.62	0.94
Retracing	0.33	0.38	-0.61	0.06	0.61
False starts	0.68	-0.18	-0.38	0.27	0.67
Target unclear	0.37	-0.05	-0.05	0.86	0.86
Meaning unclear	0.48	0.54	0.42	-0.01	0.89
Apraxia of speech	-0.13	-0.18	0.06	0.88	0.73

Expressive aphasia	0.21	0.54	0.54	0.20	0.97
	Factor inter-correlations				
	Paraphasia	Logopenia	Agrammatism	Motor speech	
Paraphasia	1				
Logopenia	0.21	1			
Agrammatism	-0.09	0.24	1		
Motor speech	0.05	0.34	0.27	1	
Root mean squared deviation	.05				

Note. The factor analysis from Casilio *et al.*[1] was conducted on data from 24 individuals with chronic post-stroke aphasia. This analysis was modified to inform the optimal selection of features to create the dimension scoring system for the present study, as described in the main text. The only modification was the factor rotation, where an oblimin-rotated solution was instead used here. Oblimin, as with other oblique rotations (e.g., geomin), permits the inter-correlation of factors whereas orthogonal rotations (e.g., varimax) constrain the loading matrix to possess as small of inter-correlations as possible. Oblique rotations are widely recommended for parsing the dimensionality of behavioral measures of cognitive processing, given that most measures tend to inter-correlate, as was the case for APROCSA features in our Casilio *et al.*[1] paper (see Fig. 3). The correlations of this oblique rotation, as shown above, are among the latent factors and thus are not expected to align perfectly with those obtained from another scaling system, such as the one reported in the main text (see Fig. 1D for inter-correlations based on our dimension scoring system). The eigenvalues associated with each factor are 8.70, 5.60, 2.41, and 1.74. Notably, the factor rotation does not influence the number of factors extracted; thus, re-review of the factor structure more generally was not conducted. This modified analysis was carried out using R 4.3.0.

References

1. Casilio M, Rising K, Beeson PM, Bunton K, Wilson SM. Auditory-perceptual rating of connected speech in aphasia. *Am J Speech Lang Pathol*. 2019;28(2):550-568.

Supplementary Table 2 Results detailing the effect of an ROI on the multivariate distribution of all four APROCSA dimension scores

	Pillai's Trace		*F*-test		
Variable	*V*	*df*	Approximate *F*	*df*	*p*
(Intercept)	.827	1	131.089	4, 110	< .001
Prefrontal ROI	.141	1	4.502	4, 110	.002
Frontoparietal ROI	.202	1	6.974	4, 110	< .001
Temporal ROI	.209	1	7.285	4, 110	< .001
Other ROI	.018	1	0.500	4, 110	.736

df = degrees of freedom

Supplementary Figure 1 APROCSA dimension scoring process

STEP 1: OBTAIN A LANGUAGE SAMPLE

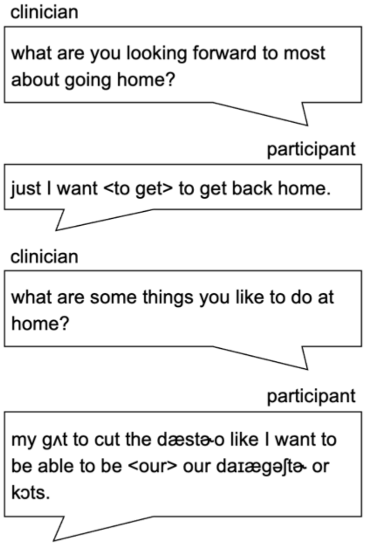

STEP 2: RATE APROCSA FEATURES

Feature	Rating
Anomia	2
Abandoned utterances	0
Empty speech	4
Semantic paraphasias	1
Phonemic paraphasias	2
Neologisms	3
Jargon	4
Perseverations	0
Stereotypies	0
Short and simplified	1
Omission of bound morphemes	0
Omission of function words	0
Paragrammatism	2
Pauses between utterances	1
Pauses within utterances	0
Halting and effortful	0
Reduced speech rate	0
Retracing	1
False starts	1
Target unclear	0
Meaning unclear	4
Expressive aphasia	3
Apraxia of speech	0

STEP 3: TRANSFORM RATINGS

Feature	Rating
Anomia	50
Abandoned utterances	0
Empty speech	100
Semantic paraphasias	25
Phonemic paraphasias	50
Neologisms	75
Jargon	100
Perseverations	0
Stereotypies	0
Short and simplified	25
Omission of bound morphemes	0
Omission of function words	0
Paragrammatism	50
Pauses between utterances	25
Pauses within utterances	0
Halting and effortful	0
Reduced speech rate	0
Retracing	25
False starts	25
Target unclear	0
Meaning unclear	100
Expressive aphasia	75
Apraxia of speech	0

STEP 4: AVERAGE THE TRANSFORMED RATINGS FOR FEATURE SETS COMPRISING THE FOUR DIMENSIONS

Feature	Paraphasia	Logopenia	Agrammatism	Motor speech
Anomia	—	50	—	—
Abandoned utterances	0	0	—	—
Empty speech	100	—	—	—
Semantic paraphasias	25	—	—	—
Phonemic paraphasias	50	—	—	—
Neologisms	75	—	—	—
Jargon	100	—	—	—
Perseverations	—	0	—	—
Stereotypies	—	—	0	—
Short and simplified utterances	—	—	25	—
Omission of bound morphemes	—	—	0	—
Omission of function words	—	—	0	—
Paragrammatism	50	—	—	—
Pauses between utterances	—	25	—	25
Pauses within utterances	—	0	—	—
Halting and effortful	—	—	—	0
Reduced speech rate	—	0	—	0
Retracing	—	—	-25	—
False starts	25	—	—	—
Target unclear	—	—	—	0
Meaning unclear	100	100	100	—
Expressive aphasia	—	75	75	—
Apraxia of speech	—	—	—	0
**Dimension score**	58.333	31.25	25	5

Overview of the steps involved in deriving the four APROCSA dimension scores of the present study. Transcription and rating examples are from PID 1552. Untransformed APROCSA ratings are expressed on a 0–4 scale, with higher scores denoting greater impairment (see Fig. 1B for scale definitions). As noted in for Fig. 1B in the main text and following results reported in Supplementary Table 1, *Retracing* is expressed as a negative value. The following transcription conventions were used for displaying the language sample excerpt: < > indicate retracing; words transcribed using the international phonetic alphabet indicate phoneme-based errors (e.g., phonemic paraphasias, neologisms). Of note, transcription was not used in the scoring of APROCSA and is displayed here only for illustrative purposes.

4 Aspects of Talking in Aphasia after Stroke

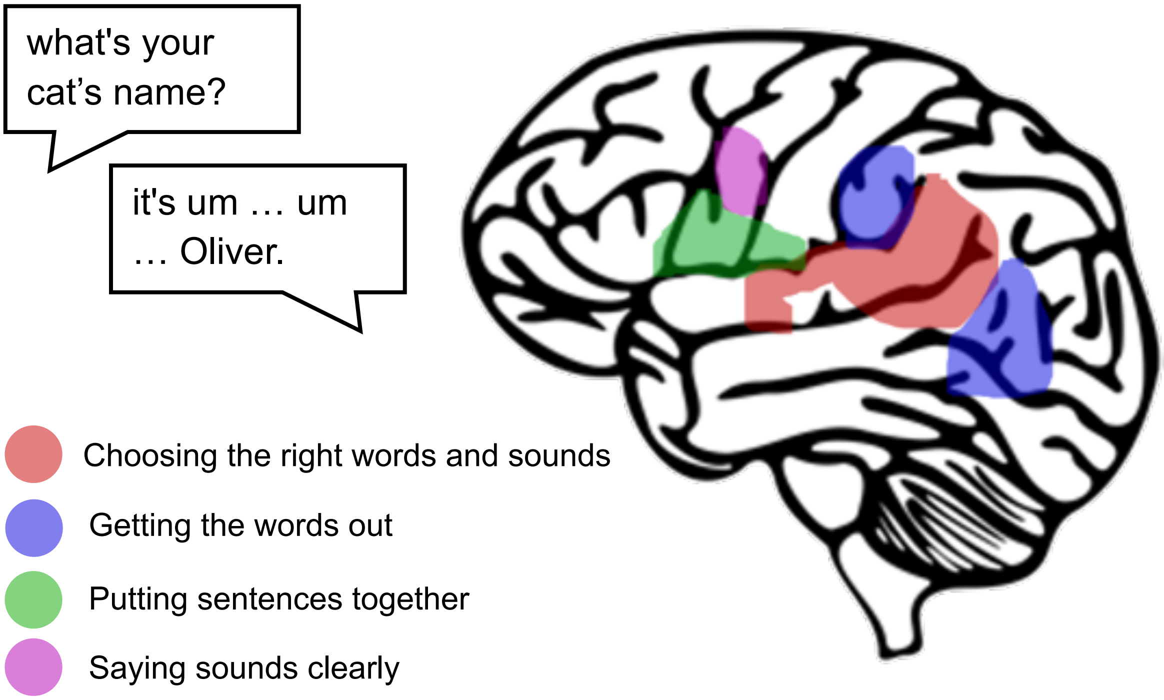

By Marianne Casilio, Anna V. Kasdan, Katherine Bryan, Kiiya Shibata, Sarah M. Schneck, Deborah F. Levy, Jillian L. Entrup, Caitlin Onuscheck, Michael de Riesthal, and Stephen M. Wilson

An accessible version of “Four dimensions of naturalistic language production in aphasia after stroke”

Abstract:
Study summary

- We wanted to better **understand how people with aphasia talk** in the **real world**

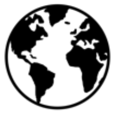

- We asked **lots of people with aphasia** after a **left-sided stroke** to do a study…

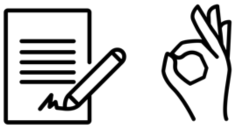

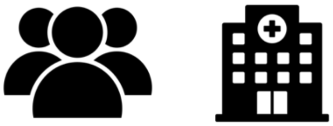

  - We looked at how **talking** was **related** to **stroke location** and **size**

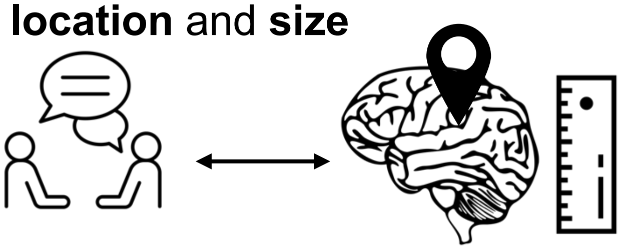

- We found that **4 aspects of talking in aphasia** were related to **4 unique areas in the brain**

Choosing the right words and sounds (Paraphasia)

Getting the words out (Logopenia)

Putting sentences together (Agrammatism)

Saying sounds clearly (Motor speech)

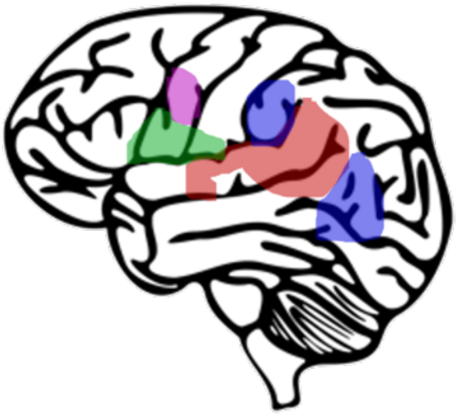

- We hope this information helps clinicians **teach patients**, **track recovery**, and **design treatments**

Introduction:
Why this study

- In research studies, people with aphasia often **name pictures**

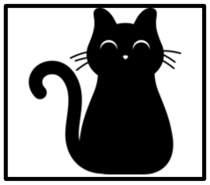

it’s a … cat.

- Or **repeat words**

say “cat.”

um … “cat.”

- But this is ***not*** **how people talk** in the **real world**
- We **know little** about how people **talk in everyday life**

what's your cat’s name?

it's um … um … Oliver.

very cool.

yeah … had him … um for t- t- t- two no ten years.

We wanted to better understand talking abilities in people with aphasia

Introduction:
Study overview

- 118 people with aphasia completed our study

- Our study involved:

    - **Measuring** everyone's **talking** in the **first week after stroke** to the **left side** of the **brain**

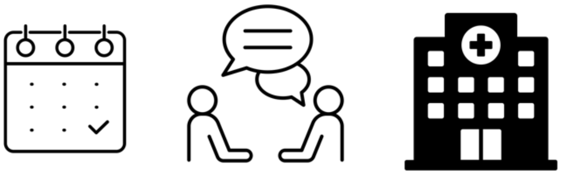

    - **Measuring** everyone's **stroke location** and **size**

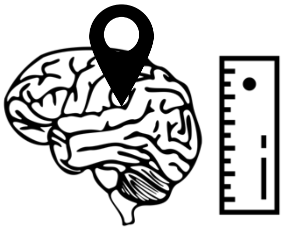

    - Analyzing **how talking related to stroke location** and **size**

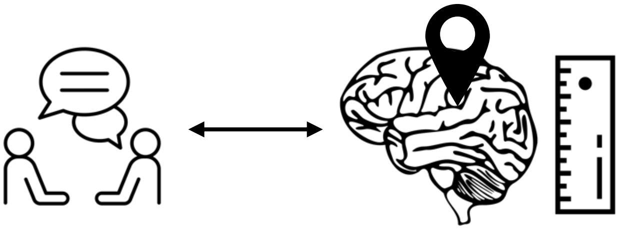

Materials and Methods:
How we measured talking (1/4)

- First, we **got permission** from people to **be in the study**

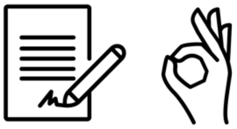

- Then, we **talked to people** about things like…
    - Family and friends 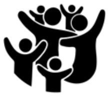
    - Work 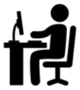
    - Hometown 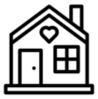
    - Hobbies 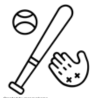
    - Favorite memories 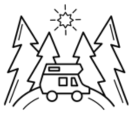
- We **video-recorded** the conversation 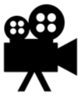
- We **watched** the **first three minutes** of people talking

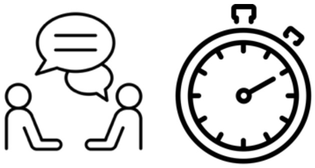

Materials and Methods:
How we measured talking (2/4)

- We evaluated talking using a tool called…

APROCSA (uh – prok – suh)

- APROCSA measures **many parts of talking** such as…
  - Pausing before saying a word

    it's a … cat.

  - Saying shorter sentences

    had him ten years.

  - Saying the first parts of words many times

    t- t- t- two

- APROCSA scoring has **2 steps**

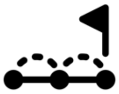

- First, we **rate** different parts of **talking** on a **5-point scale**

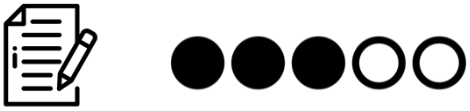

Materials and Methods:
How we measured talking (3/4)

- Second, we use the ratings to get **4 scores**

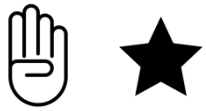

- These **4 scores** measure the **different ways** people with aphasia may **struggle with talking**

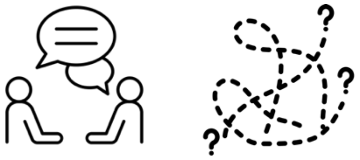

- For example, someone may want to say…

I bought an apple at the store.

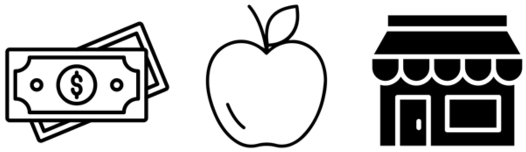

- **People with aphasia** may **struggle** to say this in **4 different ways** (the 4 scores we get)…
    - They may have difficulty…
        - Choosing the right words and sounds

I bought a ba- banana no I bought an ap- apple at oh that place.

We call this Paraphasia

Materials and Methods:
How we measured talking (4/4)

- Getting the words out

> I bought … um … I don’t know …

We call this Logopenia

- Putting sentences together

> buy … apple … store.

We call this Agrammatism

- Saying sounds clearly

> I bought .. an ap-ple .. xx ssstore.

We call this Motor speech

Materials and Methods:
How we measured stroke location and size

- First, we got **permission** to **look** at people's **medical records**

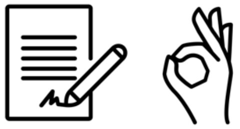

- Then, we **downloaded** people's **brain scans**

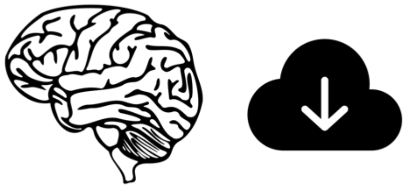

- We used **software** to **highlight** where the **stroke** was located

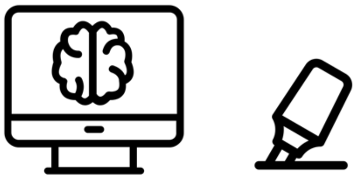

- We wrote code to **count** how much **damage** was in **different parts of the brain**

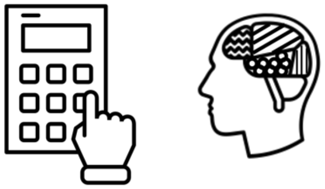

Materials and Methods:
How we analyzed the data

- We **correlated** our **4 talking scores** with **stroke location** and **size**

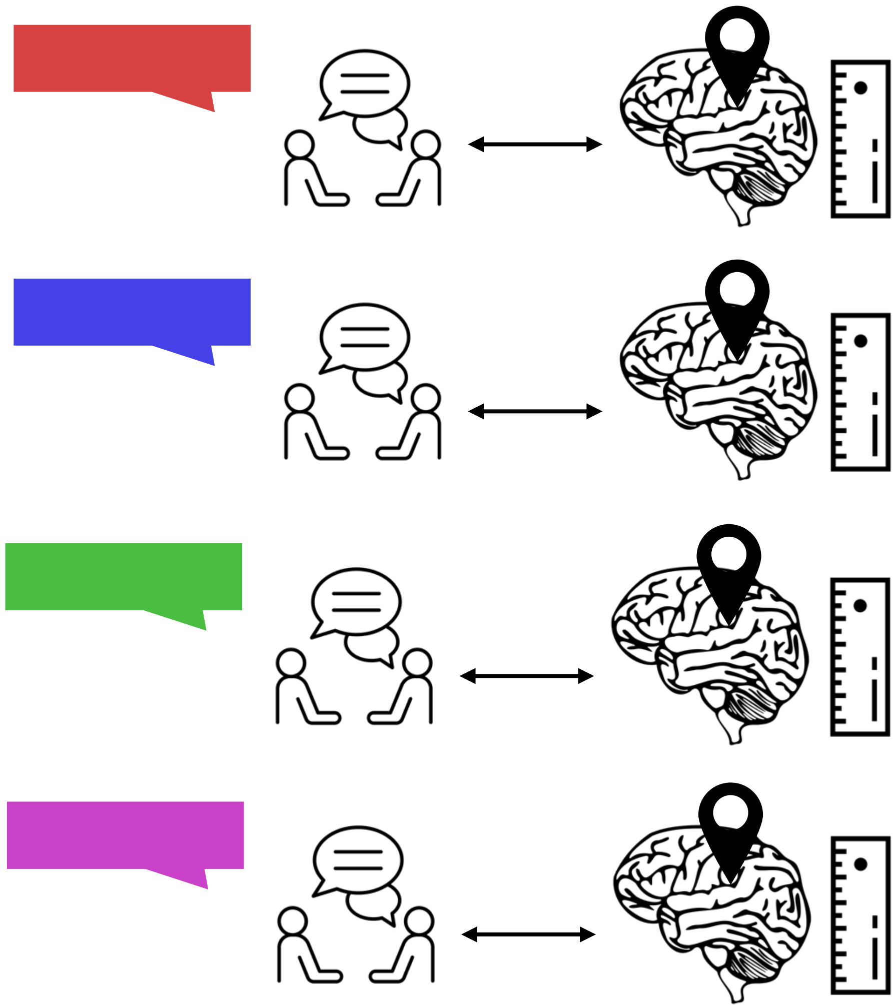

- We looked at which **stroke locations** were **unique** to our **4 talking scores**
    - We also looked at which **stroke locations** were **shared** among our **4 talking scores**

Results:
Paraphasia

- Strokes in parts of the **left temporal and parietal lobes** make it difficult to…
  - Choose the right words and sounds

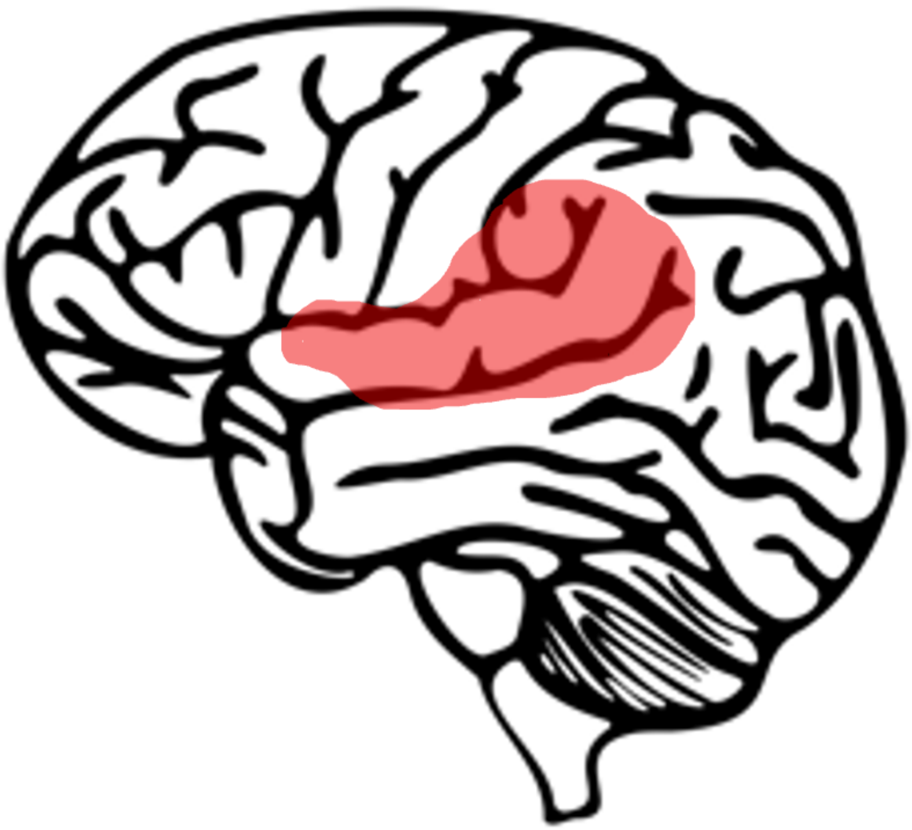

Results:
Logopenia

- Strokes primarily in parts of the **left frontal**, **left parietal**, and **left temporal lobes** make it difficult to…
    - Get the words out

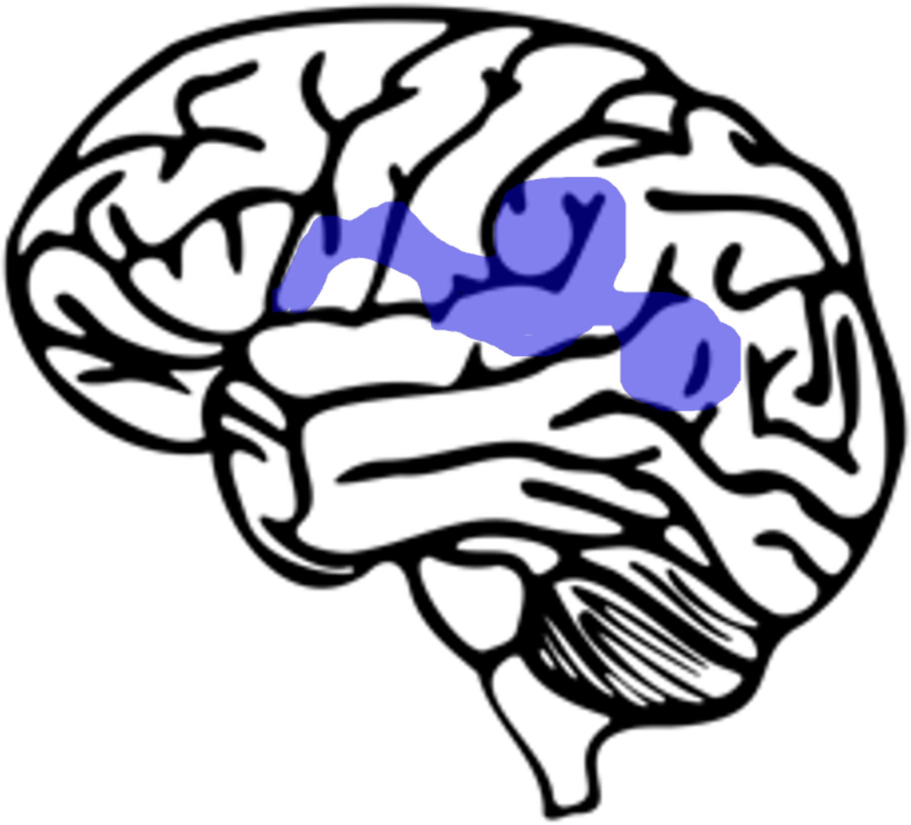

Results:
Agrammatism

- Strokes primarily in parts of the **left frontal** and **left parietal lobes** make it difficult to…
  - Put sentences together

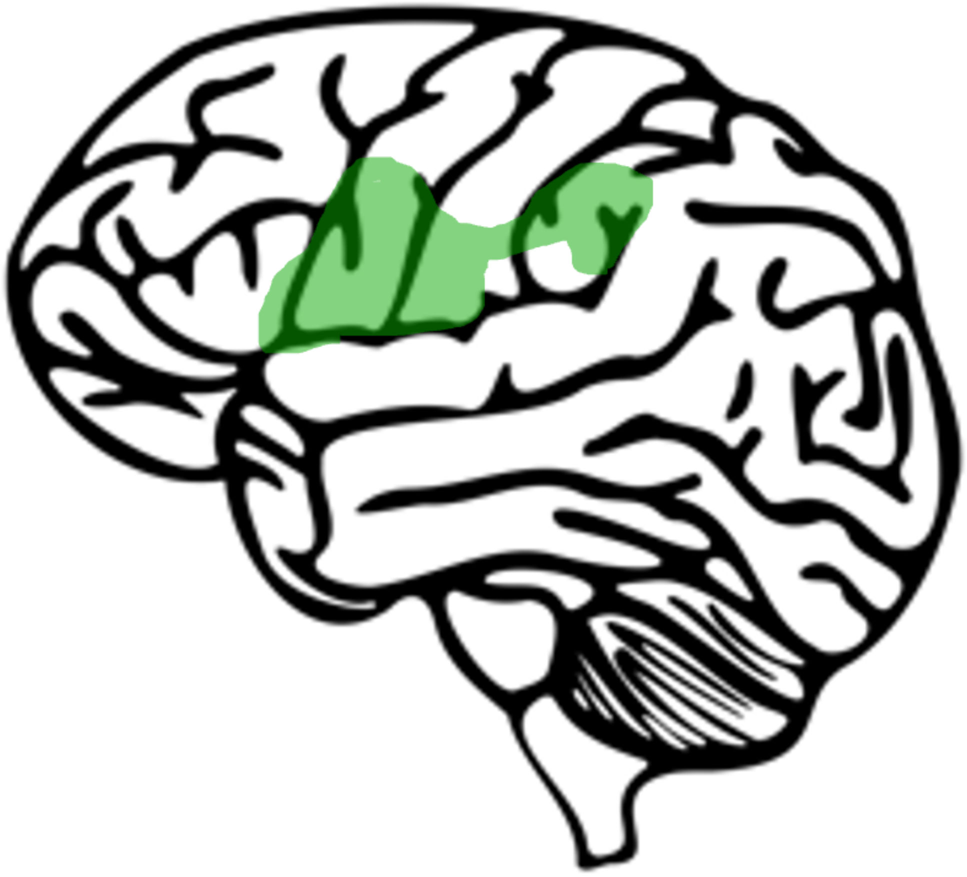

Results:
Motor speech

- Strokes primarily in the **left frontal lobe** and **left basal ganglia** (not pictured) make it difficult to…
    - Say sounds clearly

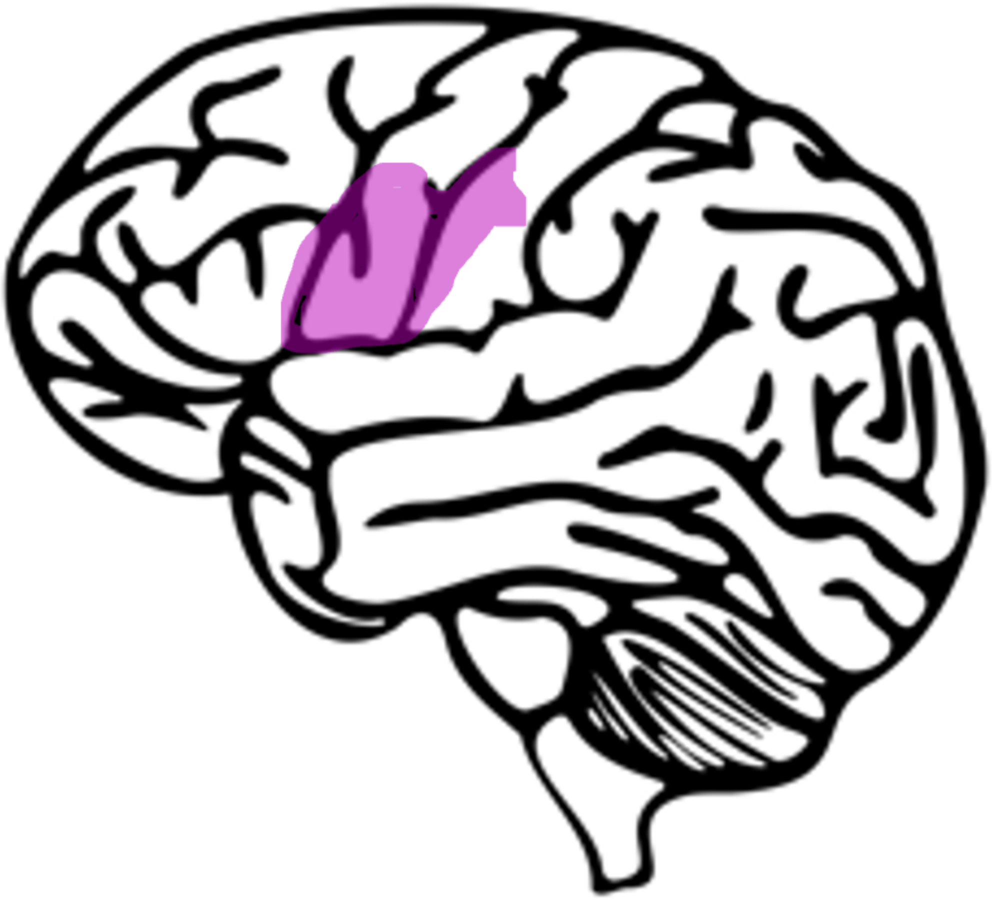

Discussion:
Main ideas (1/2)

- Overall, the **location** of the **stroke** is **more important** than the **size** when it comes to talking difficulties in aphasia in the first week after stroke

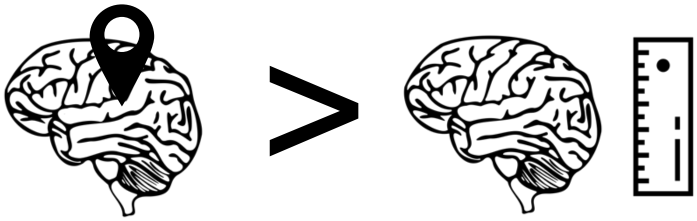

- **Many unique parts** of the **left side** of the **brain** are important for **different** aspects of **talking**…
  - Strokes located in areas toward the **back of the brain** may cause different types of **word finding difficulties**

Choosing the right words and sounds (Paraphasia)

Getting the words out (Logopenia)

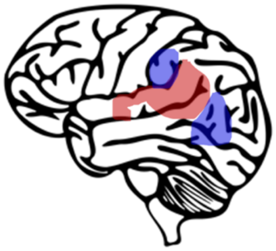

  - Strokes located in areas toward the **front of the brain** may cause difficulties with **grammar** and **speech clarity**

Putting sentences together (Agrammatism)

Saying sounds clearly (Motor speech)

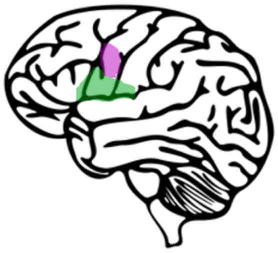

Discussion:
Main ideas (2/2)

- **2 shared parts** of the **left side** of the **brain** are important for **multiple** aspects of **talking**…
  - Strokes located in the **left frontal lobe** affect some **word finding difficulties**, **grammar**, and **speech clarity**

Getting the words out (Logopenia)

Putting sentences together (Agrammatism)

Saying sounds clearly (Motor speech)

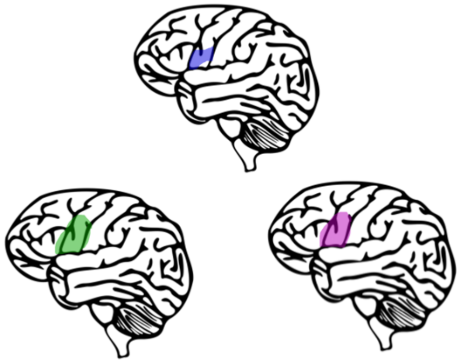

  - Strokes located in the **left parietal lobe** affects all **word finding difficulties** and **grammar**

Choosing the right words and sounds (Paraphasia)

Getting the words out (Logopenia)

Putting sentences together (Agrammatism)

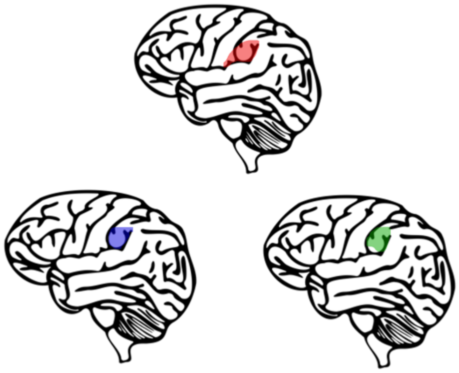

Discussion:
Why this study matters

- We designed this study to **be helpful** for **people with aphasia**, their **loved ones**, and their **care teams**

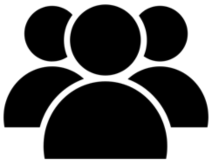 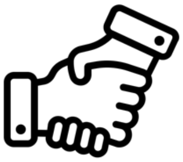

- The **information in this study** could be **used to**…
  - Teach people 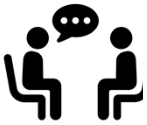
  - Track recovery 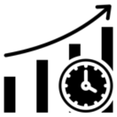
  - Design treatments 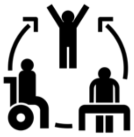

- We also have a **website for clinicians and researchers** to learn about **APROCSA**…
  - https://github.com/mcasilio/neuroaprocsa 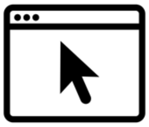

Discussion:
Limitations / Things to think about

- People who were **unable to talk for 3 minutes** were **not included** in this study…
    - We need better ways to evaluate talking in people who struggle the most

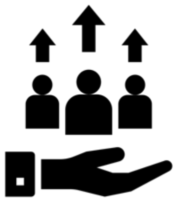

- We **did not evaluate all parts** of **talking**…
    - Building **shared meaning** and telling an **easy-to-follow story** are important and should be looked at in future studies

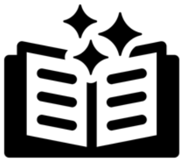

- We **did not measure thinking skills**…
    - **Memory** and **attention** may play a role in everyday talking abilities in aphasia

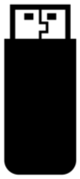

- We **did not look closely** at **connections** between **different parts of the brain**
    - **Connections** in the brain may tell us **extra information** about **everyday talking difficulties** in **aphasia**

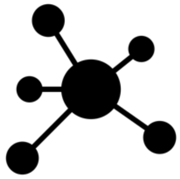

Discussion:
Conclusion

- We think we now have a **better understanding** of **how people with aphasia talk** in the **real world**

- We learned that…

    - **4 aspects of talking in aphasia** are related to **4 unique areas in the brain**

Choosing the right words and sounds (Paraphasia)

Getting the words out (Logopenia)

Putting sentences together (Agrammatism)

Saying sounds clearly (Motor speech)

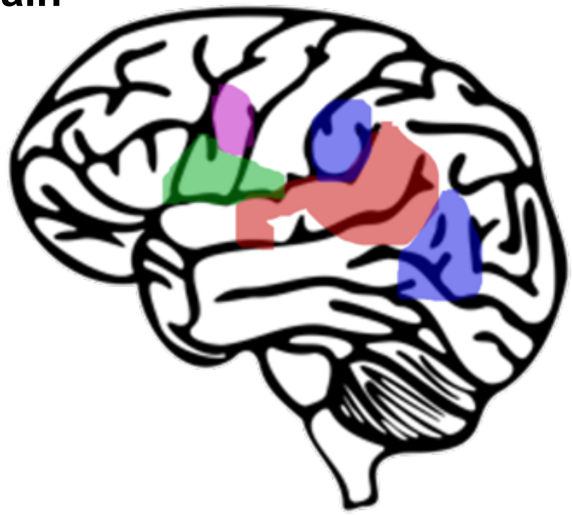

- **Thank you** for taking the time to **learn** about **this study**!
